# Identification of a Novel Neuropeptide S Receptor Antagonist Scaffold Based on the SHA-68 Core

**DOI:** 10.3390/ph14101024

**Published:** 2021-10-06

**Authors:** Allison Zarkin, Rajwana Jahan, Rajendra Uprety, Yanan Zhang, Charles McElhinny, Rodney Snyder, Elaine Gay, Gabriel Jewula, Heather Bool, Stewart D Clark, Scott Runyon

**Affiliations:** 1 Research Triangle Institute, P.O. Box 12194, Research Triangle Park, NC 27709-2194, USA; azarkin@rti.org (A.Z.); rjahan@rti.org (R.J.); ruprety@rti.org (R.U.); yzhang@rti.org (Y.Z.); cjm@rti.org (C.M.); rsnyder@rti.org (R.S.); egay@rti.org (E.G.); 2 Department of Pharmacology and Toxicology, State University of New York at Buffalo, 3435 Main Street, Buffalo, NY 14214, USA; gjjewula@buffalo.edu (G.J.); hmbool@buffalo.edu (H.B.); stewartc@buffalo.edu (S.D.C.)

**Keywords:** neuropeptide S, antagonist, anxiolytic, NPSR

## Abstract

Activation of the neuropeptide S receptor (NPSR) system has been shown to produce anxiolytic-like actions, arousal, and enhance memory consolidation, whereas blockade of the NPSR has been shown to reduce relapse to substances of abuse and duration of anesthetics. We report here the discovery of a novel core scaffold (+) N-benzyl-3-(2-methylpropyl)-1-oxo-3-phenyl-1H,3H,4H,5H,6H,7H-furo[3,4-c]pyridine-5-carboxamide with potent NPSR antagonist activity in vitro. Pharmacokinetic parameters demonstrate that **14b** reaches pharmacologically relevant levels in plasma and the brain following intraperitoneal (i.p.) administration, but is cleared rapidly from plasma. Compound **14b** was able to block NPS (0.3 nmol)-stimulated locomotor activity in C57/Bl6 mice at 3 mg/kg (i.p.), indicating potent in vivo activity for the structural class. This suggests that **14b** can serve as a useful tool for continued mapping of the pharmacological functions of the NPS receptor system.

## 1. Introduction

The neuropeptide S (NPS) system was first described publicly more than 15 years ago [1]; however, its therapeutic potential has not yet been achieved due to the lack of high-affinity drug-like ligands. Central administration of NPS (20 AA peptide) into mice produces anxiolytic-like actions, arousal, and enhances memory consolidation [1,2,3]. These wide-ranging effects are mediated by NPS receptors (NPSR) distributed throughout the CNS, e.g., cortex, amygdala, parasubiculum, and hypothalamus [4]. Expression of NPS is highly localized to discrete nuclei, e.g., peri-locus coerelus [1], although there are established interactions with the CRF (corticotropin releasing factor) system which support a role for NPS in the modulation of stress [5,6,7]. The exact connectivity of the NPSR-expressing and NPS-expressing neurons is still under investigation, and so are a number of other fundamental elements of this neuromodulatory system.

**Figure 1:**
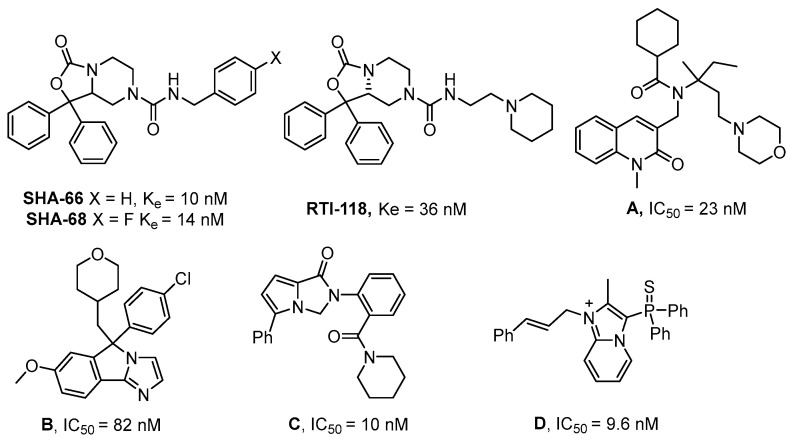
Representative NPSR antagonists.

The discovery of small-molecule NPSR agonist molecules would represent a new class of non-sedating fast-acting anxiolytics, with memory enhancement properties [3]. However, NPSR antagonists have shown promise for treating relapse to substances of abuse [5,8] and may enhance the duration of mechanistically distinct anesthetics [9]. Improved NPSR agonists would be specifically useful for elucidating aspects of the NPS-system. In addition, more drug-like NPSR antagonists would be useful as tool compounds for target validation. However, currently available NPSR antagonists (Figure 1, Compounds A–D) have good potency but tend to be highly lipophilic and have a high number of sp2 hybridized atoms. These properties result in compounds that are challenging to formulate for in vivo work [10,11,12,13,14,15].

**Figure 2:**
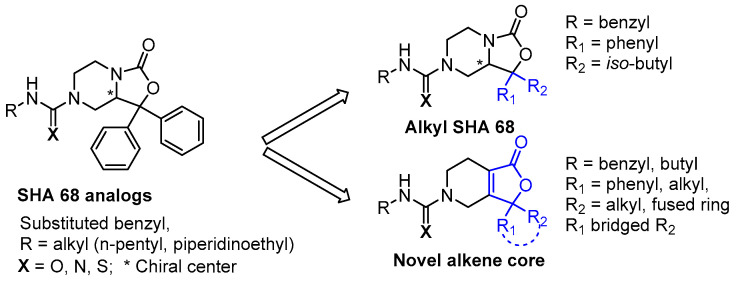
Further modifications to SHA led to the identification of novel alkene scaffolds.

For a number of known NPSR antagonists, only limited structure–activity relationship (SAR) studies have been reported. The SHA-66/68 class of molecules, which were disclosed by Okamura et al. in 2008, possess poor drug-like properties, particularly aqueous solubility, and several structural modifications have been undertaken by our group and others, resulting in a number of improved analogs such as RTI-118 [10,12]. One structural feature of the SHA class that has not been thoroughly investigated is the diaryl moiety. The chemical synthesis of SHA makes differential modification of the diaryl moiety cumbersome and introduces an additional chiral center. Considering the high sp2 count of SHA and the metabolic liabilities associated with unsubstituted aryl rings, we hypothesized that modification of this feature could lead to improved NPSR antagonists. We envisioned that replacement of the carbamate nitrogen in SHA with an alkene would provide analogs that retain the overall geometry of SHA and keep in place a number of the critical pharmacophoric elements (Figure 2). SHA already contains one chiral center; therefore, differentially substituting the diaryl core would result in a complex mixture of diastereomers that could be challenging to resolve. The addition of a double bond at the bicyclic ring junction eliminates one chiral center, leaving the remaining chiral center at the differentially substituted aryl rings of the oxazolidinone core. Therefore, our group explored structural modifications that retained the overall shape of SHA-68 yet allowed chemically tractable and differential modification of the diaryl ring system. Our resulting core, 4,5,6,7-tetrahydrofuro[3,4-c]pyridin-1(3H)-one, has produced a series of novel NPSR antagonists with similar antagonist potency in vitro, and favorable activity in vivo.

## 2. Chemistry

**Scheme 1:**
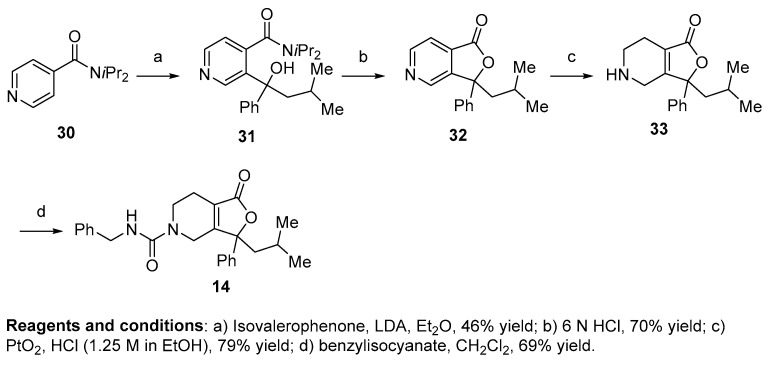
General synthetic route to alkene-based NPS antagonists.

Compounds **1**, **2**, **8**–**19**, **21**, **22**, and **24**–**28** (Table 1, Table 2 and Table 3; Figures S1–S28, Supplementary Materials) were synthesized in a manner analogous to that outlined in Scheme 1. Previous research by Epsztajn et al. [16] demonstrated that the ortho lithiation of diisopropyl nicotinamides proceeded smoothly and the resulting anion could be condensed with various ketones and aldehydes to afford the precursor to the bicyclic ring structures needed for our investigation. N,N-diisopropylisonicotinamide **30** was treated with lithium di-isopropylamide to form the anion of **30** which was condensed with the appropriate ketone to yield an alcohol intermediate, such as compound **31**, in moderate yield. Upon treatment with aqueous acid (6N HCl), the alcohol readily cyclizes into lactone **32**. A series of reduction conditions were evaluated for the pyridyl ring. We discovered reduction of the pyridine ring with 1 equivalent of acid, hydrogen, and platinum oxide as a catalyst partially reduced ring **33**, leaving the tetrasubstituted double bond intact. These reduction conditions afforded the 4,5,6,7-tetrahydrofuro[3,4-c]pyridin-1(3H)-one core **33** in good yield. Final treatment of amine **33** with the desired isocyanate in dichloromethane afforded target compounds **14a**,**b** in good yield. 

**Scheme 2:**
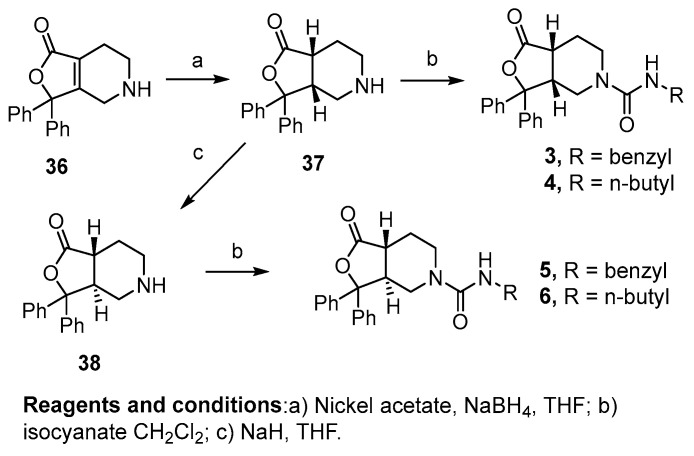
Synthesis of saturated diaryl analogs using the alkene core.

Reduction of the 4,5,6,7-tetrahydrofuro[3,4-c]pyridin-1(3H)-one core **36** with nickel acetate and sodium borohydride resulted in the reduced *cis*-intermediate **37**, which was then alkylated with benzyl or butyl isocyanate to yield compounds **3** and **4**, respectively. Prior to alkylation, the *cis*-intermediate **37** was also treated with sodium hydride to isomerize the alpha proton to the *trans*-isomer. Although this resulted in an inseparable mixture of the *cis*- and *trans*-diastereomers, after alkylation with benzyl or butyl isocyanate, the diastereomers could be separated to yield the pure *trans*-analogs **5** and **6** (Scheme 2). 

Previous work by Niiyama and colleagues [17] demonstrated that the addition of aryl Grignard reagents to pyridine 3,4-dicarboxylic acid anhydride affords compounds with selective addition of the Grignard to the carbonyl para to the pyridyl nitrogen. Compound **7** was obtained from the addition of excess phenyl magnesium bromide to pyridine 3,4-dicarboxylic acid anhydride to yield pyridine intermediate **39**. The sequence depicted in Scheme 1 was then followed to yield target compound **7**. 

**Scheme 3:**
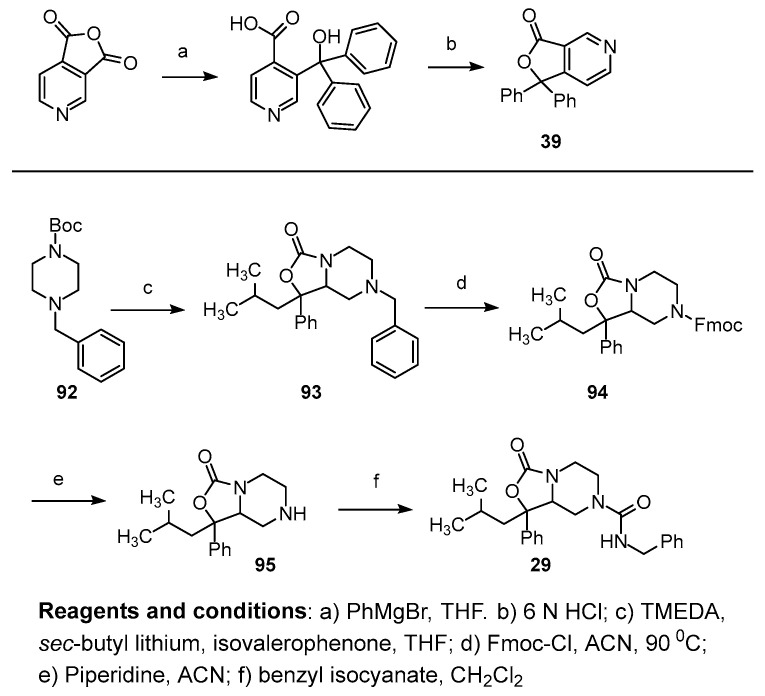
Synthesis of 3-N analogs and isobutyl replacement for phenyl in SHA.

Oxazolidinone analog **29**, which mimics SHA with one phenyl group replaced with an isobutyl group, was synthesized from N-*t*-butoxy carbonyl, N-benzyl piperidine. Deprotonation of piperidine **92** was accomplished with *sec*-butyl lithium and tetramethylene diamine. The anion was quenched with the addition of isovalerophenone followed by cyclization to the oxazolidinone ring **93**. Catalytic reduction of the benzyl ring was unsuccessful due to hydrogenolysis of the oxazolidinone ring. Removal of the benzyl group was undertaken with FMOC-chloride (**94**) followed by deprotection of the FMOC group with piperidine to yield the free amine **95**. Treatment of the free amine **95** with benzyl isocyanate yielded the desired compound **29** (Scheme 3; Figure S29, Supplementary Materials). 

## 3. Results and Discussion

We hypothesized that a 4,5,6,7-tetrahydrofuro[3,4-c]pyridin-1(3H)-one core, which is similar to that of SHA-68, would allow access to broader functional group modifications and, ultimately, allow access to a larger SAR study than those previously reported for SHA-related antagonists. Compound **1** maintained the same substituents as SHA-68 but incorporated a fused double bond in place of the oxazolidinone nitrogen atom. 

A direct comparison of compound **1** (Ke = 522 nM) with SHA-66 (Ke = 10 nM) indicated that replacement of the nitrogen atom with a double bond was detrimental for antagonist activity by roughly 50-fold (Table 1). We have previously shown that replacement of the benzyl urea on SHA with an *n*-butyl group resulted in compounds with equipotent antagonist activity but more conformational flexibility [10]. Therefore, we evaluated compound **2** with *n*-butyl urea; however, this was also reduced in activity compared to SHA by roughly 50-fold. This suggested that either the overall conformation of **1** was less tolerated by the receptor or the oxazolidinone nitrogen was a critical pharmacophore element. In order to investigate this further, reduction of the double bond to both the *cis*-isomers **3** and **4** and *trans*-isomers **5** and **6** were undertaken. The *cis*-isomers with a significantly different three-dimensional shape lacked antagonist activity, whereas the *trans*-isomers **5** and **6** were moderately potent with Ke values of 170 nM and 260 nM, respectively. We then evaluated whether shifting the urea substituent within the ring system, as shown in compound **7**, could restore activity, but this resulted in a significant loss of potency.

Importantly, this new core scaffold allows more efficient access to modification of the 3,3-diphenyl ring system without the introduction of a second chiral center (Table 2). Conformational control is an important aspect of ligand design. Reducing conformational freedom was explored by linking the two aryl rings together (compounds **8** and **9**) as well as removing an aryl ring (compound **10**) and activity was lost. In order to reduce the sp2 count for SHA, we envisioned the replacement of one aryl ring with an aliphatic group. Replacement of one phenyl ring into various aliphatic substituents yielded compounds **11**–**15**. The *n*-butyl compound **11** was moderately potent (K_e_ = 165 nM), but the *iso*-butyl compound **14** (K_e_ = 118 nM) was the most potent and was also anticipated to have an improved metabolic profile. Integrating various heteroatoms with hydrogen bond acceptors or donors into this portion of the molecule, such as a dimethylamino group or a hydroxy group (**17**–**21**), resulted in a loss of activity. In order to potentially reduce oxidative metabolism on one of the remaining unsubstituted aryl rings, we explored a series of substituted aryl rings (**22**–**25)** and found that the incorporation of naphthyl, hydroxy or benzyl ether groups (**22**–**24**) resulted in a reduction in potency. However, the 3-chlorophenyl group **25** only had a modest reduction in potency, at 136 nM compared to **14**. 

**Figure 3:**
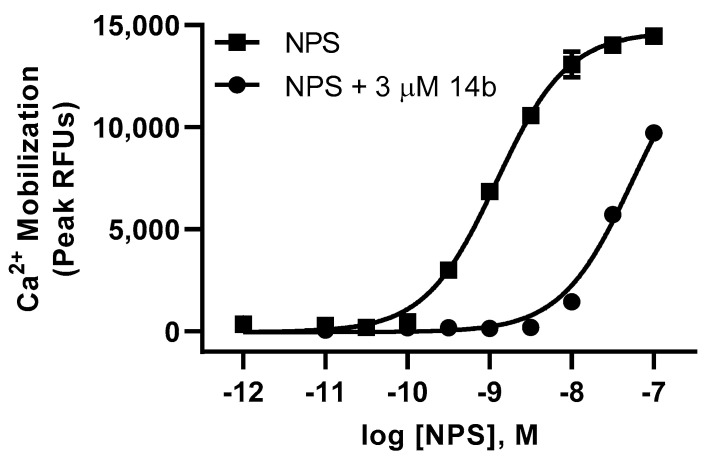
Compound **14b** displays a right shift on agonist activity of NPS in calcium mobilization assay (Ke).

Our lab has previously shown that replacement of the benzyl urea with a piperidino ethyl urea on the SHA core provides analogs with good brain permeability, enhanced solubility, and favorable in vivo activity [8,10,18]. With lead compound **14** in hand, we moved forward with exploring further modifications to the urea group using the 3-*iso*-butyl-3-phenyl substituted core (Table 3). Incorporating *n*-butyl urea (**26**), piperidino ethyl urea (**27**) and piperidino ethyl thiourea (**28**) substituents resulted in a reduction in potency. The two enantiomers of compound **14** were separated by chiral HPLC, and we determined that the plus isomer (**14b**) was the active enantiomer (K_e_ = 71.4 nM, Table 3) and showed a significant right shift to the agonist activity of NPS at 3 µM concentration in calcium mobilization assay (Figure 3). In order to evaluate if this new series was interacting with the NPSR in a similar manner as SHA, we synthesized the SHA analog with isobutyl substituted for one of the aryl rings. Compound **29** was completely inactive, suggesting that this new series binds to the NPSR in an overlapping but distinct manner.

**Scheme 4:**
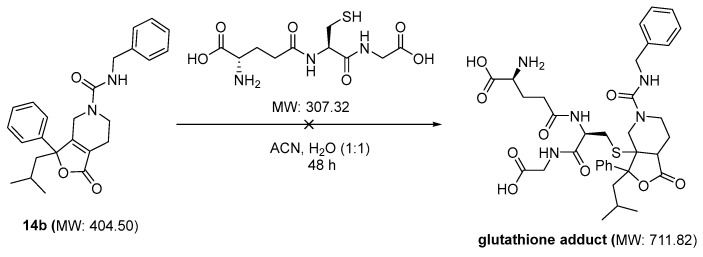
Compound **14b** is stable in the presence of excess of glutathione (18 mM).

One of the primary concerns for compound **14b** was the potential to function as a Michael acceptor and non-specifically form covalent bonds to nucleophilic proteins. We therefore evaluated the ability of glutathione to add to the alpha beta unsaturated double bond of **14b** (Scheme 4). Compound **14b** was incubated in a solution of acetonitrile and water with glutathione, according to the method of Cevher et al. [19]. Over the course of 1, 2, 4, 6, 24, and 48 h, the solution was evaluated for the appearance of molecular ions at 711 and 713 AMU. No glutathione adduct was identified over the course of the study, indicating that the tetrasubstituted double bond is most likely too sterically crowded and does not serve as an electrophilic acceptor for reactive proteins.

**Figure 4:**
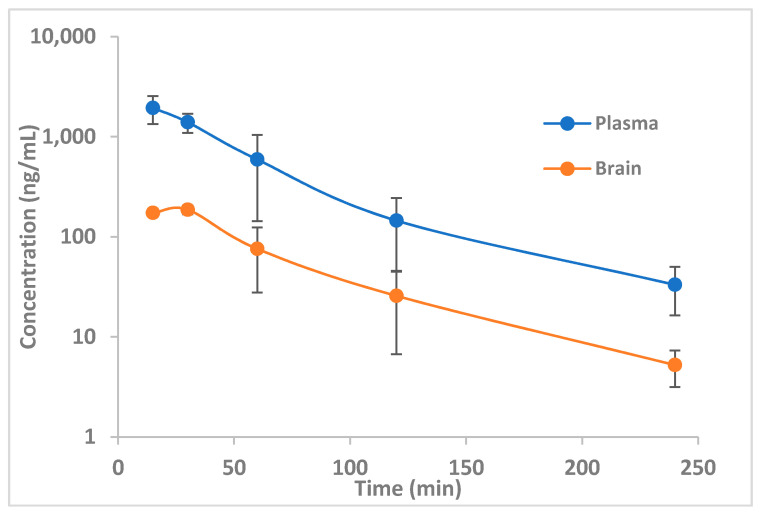
Pharmacokinetic study of **14b** in C57/Bl6 mice. Three animals/timepoint 30 mg/kg i.p. in 1% (NMP), 0.5% Tween 80, 0.5% carboxymethyl cellulose).

Considering that **14b** functions as a potent NPSR antagonist and should not serve as an electrophile in vivo, its ability to access the CNS and metabolic stability were evaluated. A pharmacokinetic study of **14b** was undertaken in C57Bl6 mice. Over the four hours following an i.p. dose of 30 mg/kg, blood and brain were collected at 15 and 30 min, followed by 1, 2, and 4 h (Figure 4). A Cmax of 1938 ng/mL and 186 ng/mL was obtained in the plasma and brain, respectively. Clearance in plasma was 288 mL/min/kg, indicating a rapid clearance most likely due to oxidative metabolism. Although the clearance was higher than desired, brain levels for the duration of behavioral experiments up to 1 h were sufficiently above the Ke of **14b**. Further optimization of metabolic stability will be addressed in future studies.

**Figure 5:**
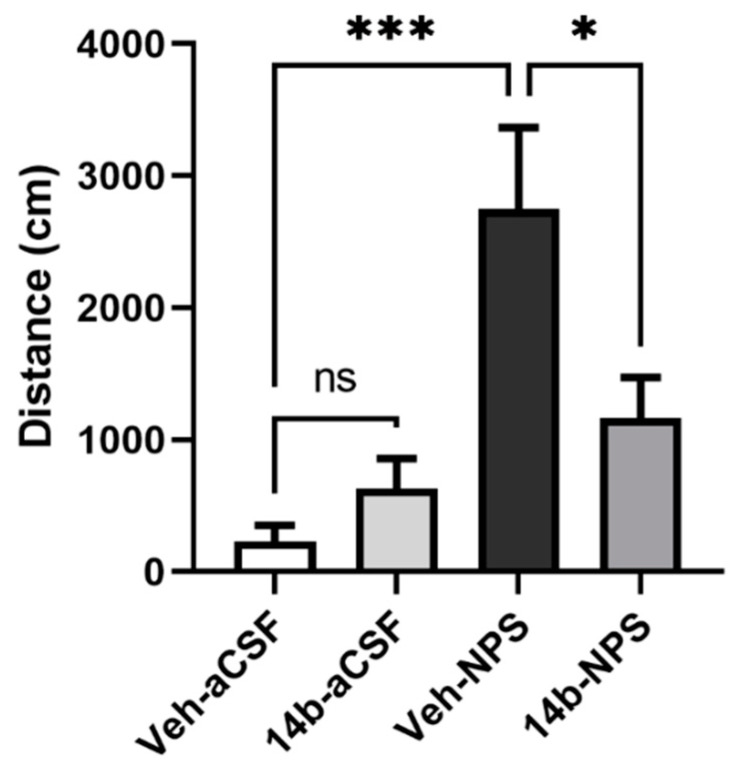
Compound **14b** attenuates the hyperlocomotory effects of NPS. After 90 min of habituation, mice were pretreated with vehicle or compound **14b** (3 mg/kg i.p.), then 10 min later, injected ICV with aCSF or NPS (0.3 nmole). Total distances traveled for the first 10 min after the ICV injection are displayed in the above graph. Compound **14b** (3 mg/kg i.p.) significantly decreased the hyperlocomotor effects of NPS. (*** *p* < 0.001, * *p* < 0.05; Veh-aCSF *n* = 6, 14b-aCSF *n* = 6, Veh-NPS *n* = 5, **14b**-NPS *n* = 4).

After establishing that sufficient levels of compound **14b** reached the CNS after systemic injection, it was evaluated in vivo for antagonist activity in a well-established NPSR-mediated behavior; NPS-mediated hyperlocomotion [1]. In this behavioral paradigm, mice are habituated to an open arena and centrally administered NPS, which has been shown to increase locomotion in a dose-dependent and NPSR-dependent manner. In the current study, this NPS-mediated effect was attenuated with the pretreatment of systemically administered 3 mg/kg compound **14b** (Figure 5; one-way ANOVA found a significant effect, *p* = 0.004, with Tukey’s post hoc analysis showing significant differences in the following comparisons: Veh-aCSF vs. Veh-NPS *p* = 0.0004, Veh-NPS vs. **14b**-aCSF *p* = 0.0020, Veh-NPS vs. **14b**-NPS *p* = 0.0389). However, compound **14b** did not decrease locomotion on its own (3 mg/kg; Veh-aCSF vs. **14b**-aCSF *p* = 0.8199). The data suggest that compound **14b** is highly effective in blocking the effects of exogenously administered NPS. The dose of NPS used in this study was one-third of that required to produce maximal effects in this paradigm; therefore, it is likely that compound **14b** had adequate potency and affinity to antagonize endogenous NPS. 

## 4. Experimental Section

All standard reagents were commercially available. Compounds were purified by column chromatography on a Teledyne Isco Rf chromatography unit and by HPLC on an Agilent-Varian HPLC system equipped with Prostar 210 dual pumps, a Prostar 335 Diode UV detector and a SEDEX75 (SEDERE, Olivet, France) ELSD detector. The HPLC solvent system was binary, water containing 0.1% trifluoroacetic acid (TFA) and solvent B (acetonitrile containing 5% water and 0.1% TFA). A semi-preparative Synergy Hydro^®^ RP 80A C18 column (4 μm 250 × 21.2 mm column; Phenomenex) was used to purify final compounds at 15 mL/min using a linear gradient from 5% to 50 or 60% B over 30 min. The purity of final compounds was determined using an analytical Synergy Hydro^®^ RP80A C18 (4 μm 250 × 4.60 mm column; Phenomenex, Torrance, CA, USA) with a linear gradient of 5–95% solvent B over 20 or 30 min at a flow rate of 1 mL/min. Absorbance was monitored at 220 nm. *Cis*/*Trans* diastereomers were separated using a semi-preparative RP HPLC YMC ODS-A (S5μm, 120 Å, 20 × 250 mm; 10 mL/min) with isocratic conditions (35:65 acetonitrile/water) at 204 nm. Diastereomeric purity was determined using an analytical RP HPLC YMC ODS-A (S5μm, 120 Å, 4.6 × 250 mm; 1 mL/min). Enantiomers of compound **14** were separated by analytical and semi-preparative chiral HPLC performed using a dual-pump system (Dynamax SD-300 solvent system delivery system with 25 mL pump heads), a Rheodyne injector and a Varian ProStar 330 diode-array detector (DAD) controlled by Varian Star Workstation software.

The molecular ion of final compounds was determined using a PE Sciex API 150 EX LC/MS system from Perkin Elmer (San Jose, California). Reactions were monitored by thin-layer chromatography (TLC) carried out on pre-coated 60 Å 250 mm silica gel TLC plates with F-254 indicator visualized under UV light, and developed using ceric ammonium molybdate. ^1^H NMR spectra were recorded at 300 MHz on a Bruker Avance 300 Spectrospin instrument and are reported as follows: chemical shift δ in ppm (multiplicity, coupling constant (Hz), and integration. The following abbreviations were used to explain multiplicities: s, singlet; d, doublet; t, triplet; q, quartet; quin, quintet; m, multiplet; br, broad; dd, doublet of doublets; dt, doublet of triplets; ddd, doublet of doublets of doublets; ddt, doublet of doublet of triplets.

### 4.1. Procedure A. 3-(1-Hydroxy-3-methyl-1-phenylbutyl)-N,N-bis(1-methylethyl)pyridine-4-carboxamide (***31***)

LDA (2.0 M, 14.5 mL, 29.1 mmol, 3 equiv.) was added to N,N-diisopropylisonicotinamide **30** (2.0 g, 9.70 mmol, 1 equiv.) at −78 °C. The reaction was stirred for 1.5 h and a solution of isovalerophenone (4.72 g, 29.1 mmol, 3 equiv.) in ether was then added and stirred for 40 min at −78 °C. The reaction mixture was quenched with water and extracted. The organic layers were combined, dried with sodium sulfate, and concentrated under reduced pressure. The resulting residue was purified by silica gel column chromatography to yield compound **31** (1.65 g, 4.48 mmol, 46% yield). ^1^H NMR (300 MHz, CDCl_3_) δ ppm 0.37 (d, *J* = 6.78 Hz, 3H), 0.57 (d, *J* = 6.78 Hz, 3H), 0.89–1.02 (m, 6H), 1.19–1.30 (m, 3H), 1.39 (d, J-6.78 Hz, 3H), 1.98–2.14 (m, 2H), 2.21–2.36 (m, 1H), 3.08–3.32 (m, 2H), 6.16 (d, *J* = 1.51 Hz, 1H), 7.14–7.41 (m, 5H), 8.54 (d, *J* = 4.90 Hz, 1H), 8.60–8.71 (m, 1H), 8.96 (s, 1H).

### 4.2. Procedure B. 3-(2-Methylpropyl)-3-phenylfuro[3,4-c]pyridin-1(3H)-one (***32***)

Compound **31** (2.2 g, 5.69 mmol) was dissolved in 6 M HCl in dioxane (40 mL). The reaction was stirred at room temperature overnight. The reaction was then basified with aqueous sodium bicarbonate, diluted with CH_2_Cl_2_, and extracted. The organic layers were combined, dried with sodium sulfate, and concentrated under reduced pressure. The resulting residue was purified by silica gel column chromatography to yield compound **32** (1.12 g, 4.19 mmol, 74% yield). ^1^H NMR (300 MHz, CDCl_3_) δ ppm 0.86 (dd, *J* = 11.68, 6.78 Hz, 6H), 1.47–1.71 (m, 1H), 1.99–2.14 (m, 1H), 2.52 (dd, *J* = 14.88, 5.09 Hz, 1H), 7.27–7.46 (m, 3H), 7.48–7.61 (m, 2H), 7.76 (dd, *J* = 4.90, 1.13 Hz, 1H), 8.83 (d, *J* = 4.90 Hz, 1H), 9.04 (d, *J* = 1.13 Hz, 1H).

### 4.3. Procedure C. 3-(2-Methylpropyl)-3-phenyl-4,5,6,7-tetrahydrofuro[3,4-c]pyridin-1(3H)-one (***33***)

Compound **32** (1.12 g, 4.19 mmol, 1 equiv.) was dissolved in 1.25 M HCl in ethanol (3.35 mL, 1 equiv.). A catalytic amount of PtO_2_ was added and the mixture was put under hydrogen on a parr hydrogenator at 40 psi for 4 h. The PtO_2_ was removed by filtration and the solvent was removed under reduced pressure. EtOAc was added to the residue, and it was basified with aqueous sodium bicarbonate and extracted. The organic layer was dried with sodium sulfate and concentrated under reduced pressure, and the residue was purified by silica gel column chromatography to yield alkene **33** (0.90 g, 3.32 mmol, 79% yield). ^1^H NMR (300 MHz, CDCl_3_) δ ppm 0.77–1.01 (m, 6H), 1.56–1.71 (m, 1H), 1.73–1.84 (m, 1H), 1.96–2.14 (m, 1H), 2.15–2.40 (m, 3H), 2.81–3.06 (m, 2H), 3.40–3.51 (m, 1H), 3.59–3.74 (m, 1H), 7.20–7.44 (m, 5H). ESI MS *m*/*z*: Calculated for C_17_H_21_NO_2_ 271.16, Found 270.2 (M-H)^-^.

### 4.4. Procedure D

#### 4.4.1. N-Benzyl-3-(2-methylpropyl)-1-oxo-3-phenyl-1H,3H,4H,5H,6H,7H-furo[3,4-c]pyridine-5-carboxamide (**14a**,**b**)

Benzyl isocyanate (0.029 g, 0.22 mmol, 1.2 equiv.) was added to compound **33** (0.05 g, 0.184 mmol, 1 equiv.) in CH_2_Cl_2_ (10 mL). The reaction was stirred at room temperature for 1 h. The reaction mixture was concentrated under reduced pressure and the resulting reside was purified via silica gel chromatography (20% EtOAc/hexane) to yield compound **14** (0.051 g, 0.13 mmol, 69% yield). ^1^H NMR (300 MHz, CDCl_3_) δ ppm 0.85 (d, *J* = 6.40 Hz, 3H), 0.89–0.98 (m, 3H), 1.65 (ddd, *J* = 13.56, 6.78, 4.90 Hz, 1H), 1.73–1.87 (m, 1H), 2.24–2.40 (m, 3H), 3.27 (ddd, *J* = 13.56, 7.35, 5.09 Hz, 1H), 3.54 (dt, *J* = 13.66, 5.23 Hz, 1H), 3.92 (dt, *J* = 18.84, 2.64 Hz, 1H), 4.40 (d, *J* = 5.65 Hz, 2H), 4.49 (dt, *J* = 19.12, 2.12 Hz, 1H), 5.00 (t, *J* = 5.46 Hz, 1H), 7.21–7.42 (m, 10H). The separation of the enantiomers of compound **14** was achieved by semi-preparative chiral HPLC performed on an amylose-based chiral column (Chiralpak IA 250 × 20 mm, 5 µm). An isocratic elution employing hexanes with 0.1% diethylamine /isopropanol with 0.1% diethylamine (90:10) at a flow rate of 15 mL/min and following by UV (240 nm) afforded the purified enantiomers. HPLC analysis (Chiralpak IA, 4.6 × 250 mm, 5 μM, hexanes containing 0.1% diethylamine/isopropanol containing 0.1% diethylamine (90:10), 1.0 mL/min, 240 nm) indicated that peak 1 (8.760 min) was >99% ee (did not detect the other enantiomer) [α]_D_^22^ = −60.9 (c 0.96, MeOH) and peak 2 (10.067 min) was 96.2% ee. [α]_D_^22^ = +61.9 (c 1.03, MeOH).

#### 4.4.2. N-Benzyl-1-oxo-3,3-diphenyl-1H,3H,4H,5H,6H,7H-furo[3,4-c]pyridine-5-carboxamide (**1**)

Benzyl isocyanate was reacted with **36** according to procedure D to yield compound **1** (0.031 g, 0.073 mmol, 86% yield). ^1^H NMR (300 MHz, CDCl_3_)δ ppm 2.47 (tt, *J* = 5.46, 2.45 Hz, 2H), 3.51 (t, *J* = 5.65 Hz, 2H), 4.27 (t, *J* = 2.45 Hz, 2H), 4.41 (d, *J* = 5.65 Hz, 2H), 4.97 (t, *J* = 5.27 Hz, 1H), 7.18–7.32 (m, 8H), 7.31–7.40 (m, 7H); MS (ESI) *m*/*z*: calcd for C_27_H_24_N_2_O_3_ 424.49, found 425.4 [M + H]^+^. HPLC (220 nm) *t*_R_ = 15.68 min. 

#### 4.4.3. N-Butyl-1-oxo-3,3-diphenyl-1H,3H,4H,5H,6H,7H-furo[3,4-c]pyridine-5-carboxamide (**2**)

Butyl isocyanate was reacted with **36** according to procedure D to yield compound **2** (0.024 g, 0.061 mmol, 72% yield). ^1^H NMR (300 MHz, CDCl_3_) δ ppm 0.85–0.97 (m, 3H), 1.18–1.54 (m, 4H), 2.49 (tt, *J* = 5.46, 2.45 Hz, 2H), 3.23 (td, *J* = 7.06, 5.46 Hz, 2H), 3.50 (t, *J* = 5.65 Hz, 2H), 4.25 (t, *J* = 2.45 Hz, 2H), 4.55 (t, *J* = 5.46 Hz, 1H), 7.20–7.31 (m, 4H), 7.33–7.41 (m, 6H). MS (ESI) *m*/*z*: calcd for C_24_H_26_N_2_O_3_ 390.47, found 391.4 [M + H] ^+^. HPLC (220 nm) *t*_R_ = 15.63 min.

#### 4.4.4. (3,7)-N-Benzyl-1-oxo-3,3-diphenyl-octahydrofuro[3,4-c]pyridine-5-carboxamide (**3** and **5**)

The mixture of *cis* and *trans* amines 37 and 38 was reacted with benzyl isocyanate according to procedure D. The mixture was then separated by HPLC to yield *cis* isomer compound **3** and *trans* isomer 5. ^1^H NMR (300 MHz, CDCl_3_) δ ppm 7.54 (d, *J* = 7.7 Hz, 2H), 7.44 (d, *J* = 7.7 Hz, 2H), 7.39–7.18 (m, 11H), 4.79–4.68 (m, 1H), 4.48–4.31 (m, 2H), 3.94 (dd, *J* = 14.1, 5.8 Hz, 1H), 3.64–3.49 (m, 2H), 2.96–2.78 (m, 2H), 2.38–2.24 (m, 1H), 2.10 (d, *J* = 13.7 Hz, 1H), 1.93–1.75 (m, 1H); MS (ESI) *m*/*z*: calcd for C_27_H_26_N_2_O_3_ 426.51, found 427.4 [M + H]^+^. HPLC (220 nm) *t*_R_ = 15.80 min. Trans isomer 5 HPLC (220 nm) *t*_R_ = 15.89 min.

#### 4.4.5. (3,7)-N-Butyl-1-oxo-3,3-diphenyl-octahydrofuro[3,4-c]pyridine-5-carboxamide (**4**)

*Cis* amine **37** was reacted with butyl isocyanate according to procedure D to yield compound **4** (0.031 g, 0.079 mmol, 92% yield). ^1^H NMR (300 MHz, CDCl_3_) δ ppm 0.92 (t, *J* = 7.35 Hz, 3H), 1.24–1.52 (m, 4H), 1.75–1.92 (m, 1H), 2.10 (d, *J* = 13.94, 1H), 2.28 (dd, *J* = 14.13, 11.87, 1H), 2.77–2.96 (m, 2H), 3.11–3.28 (m, 2H), 3.47–3.62 (m, 2H), 3.91 (dd, *J* = 14.32, 5.65 Hz, 1H), 4.40 (t, *J* = 5.27 Hz, 1H), 7.17–7.38 (m, 6H), 7.42–7.48 (m, 2H), 7.50–7.59 (m, 2H); MS (ESI) *m*/*z*: calcd for C_24_H_28_N_2_O_3_ 392.49, HPLC (220 nm) *t*_R_ = 16.42 min.

#### 4.4.6. (3,7)-N-Butyl-1-oxo-3,3-diphenyl-octahydrofuro[3,4-c]pyridine-5-carboxamide (**6**)

The isomerized mixture of *cis* and *trans* amines 37 and 38 was reacted with butyl isocyanate according to procedure D. The mixture of isomers was separated by HPLC to yield compound **6** (0.036 g, 0.095 mmol, 35% yield). ^1^H NMR (300 MHz, CDCl_3_) δ ppm 0.94 (t, *J* = 7.16, 3H), 1.30–1.71 (m, 6H), 2.10–2.43 (m, 2H), 2.59–2.90 (m, 2H), 3.16–3.38 (m, 2H), 3.74 (d, *J* = 12.43 Hz, 1H), 4.49 (br.s, 1H), 4.96 (d, *J* = 10.55 Hz, 1H), 7.12–7.22 (m, 2H), 7.28–7.44 (m, 6H), 7.51–7.59 (m, 2H); MS (ESI) *m*/*z*: calcd for C_24_H_28_N_2_O_3_ 392.49, found 393.4 [M + H]^+^. HPLC (220 nm) *t*_R_ = 3.21 min.

#### 4.4.7. N-Benzyl-3-oxo-1,1-diphenyl-1H,3H,4H,5H,6H,7H-furo[3,4-c]pyridine-5-carboxamide (**7**)

Amine **40** was reacted with benzyl isocyanate according to procedure D to yield compound **7** (0.038 mg, 0.09 mmol, 52% yield). ^1^H NMR (300 MHz, CDCl_3_) δ ppm 2.48 (dt, *J* = 5.27, 2.64 Hz, 2H), 3.68 (t, *J* = 5.46 Hz, 2H), 4.09–4.16 (m, 2H), 4.41 (d, *J* = 5.27 Hz, 2H), 5.03–5.14 (m, 1H), 7.13–7.33 (m, 9H), 7.34–7.42 (m, 6H); MS (ESI) *m*/*z*: calcd for C_27_H_24_N_2_O_3_ 424.49, found 425.4 [M + H]^+^. HPLC (220 nm) *t*_R_ = 15.74 min.

#### 4.4.8. N-Benzyl-3′-oxo-4′,5′,6′,7′-tetrahydro-3′H-spiro[fluorene-9,1′-furo[3,4-c]pyridine]-6′-carboxamide (**8**)

Amine **43** was reacted with benzyl isocyanate according to procedure D to yield compound **8** (0.032 g, 0.076 mmol, 88% yield). ^1^H NMR (300 MHz, CDCl_3_) δ ppm 2.61 (tt, *J* = 5.46, 2.45 Hz, 2H), 3.53–3.62 (m, 4H), 4.33 (d, *J* = 5.27 Hz, 2H), 4.59 (t, *J* = 5.27 Hz, 1H), 7.17–7.34 (m, 9H), 7.45 (td, *J* = 7.44, 1.32 Hz, 2H), 7.65–7.75 (m, 2H); MS (ESI) *m*/*z*: calcd for C_27_H_22_N_2_O_3_ 422.48, found 423.4 [M + H]^+^. HPLC (220 nm) *t*_R_ = 15.48 min.

#### 4.4.9. N-Benzyl-3-oxo-4,5,6,7-tetrahydro-3H-spiro[furo[3,4-c]pyridine-1,2′-tricyclo[9.4.0.0^{3,8}]pentadecane]-1′(11′),3′,5′,7′,12′,14′-hexaene-6-carboxamide (**9**)

Amine **46** was reacted with benzyl isocyanate according to procedure D to yield compound **9** (0.048 g, 0.11 mmol, 79% yield). ^1^H NMR (300 MHz, CDCl_3_) δ ppm 2.49 (tt, *J* = 5.32, 2.59 Hz, 2H), 3.05–3.19 (m, 4H), 3.44 (t, *J* = 5.65 Hz, 2H), 4.08–4.12 (m, 2H), 4.34 (d, *J* = 5.27 Hz, 2H), 4.85 (t, *J* = 5.27, 1H), 7.12–7.34 (m, 13H); MS (ESI) *m*/*z*: calcd for C_29_H_26_N_2_O_3_ 450.53, found 451.4 [M + H]^+^. HPLC (220 nm) *t*_R_ = 16.09 min.

#### 4.4.10. N-Benzyl-3′-oxo-4′,5′,6,6′,7,7′,8,9-octahydro-3′H-spiro[-benzo[7]annulene-5,1′-furo[3,4-c]pyridine]-6′-carboxamide (**10**)

Amine **49** was reacted with benzyl isocyanate according to procedure D to yield compound **10** (0.071 g, 0.18 mmol, 71% yield). ^1^H NMR (300 MHz, CDCl_3_) δ ppm 1.62–1.77 (m, 1H), 1.79–2.11 (m, 4H), 2.12–2.27 (m, 1H), 2.35–2.44 (m, 2H), 2.78–2.92 (m, 1H), 2.99–3.12 (m, 1H), 3.53 (td, *J* = 5.56, 2.07 Hz, 2H), 4.18–4.32 (m, 1H), 4.33–4.45 (m, 3H), 5.14 (t, *J* = 5.46, 1H), 6.91–6.98 (m, 1H), 7.07–7.38 (m, 8H); MS (ESI) *m*/*z*: calcd for C_25_H_26_N_2_O_3_ 402.49, found 403.4 [M + H]^+^. HPLC (220 nm) *t*_R_ = 15.33 min. 

#### 4.4.11. N-Benzyl-3-butyl-1-oxo-3-phenyl-1H,3H,4H,5H,6H,7H-furo[3,4-c]pyridine-5-carboxamide (**11**)

Amine **52** was reacted with benzyl isocyanate according to procedure D to yield compound **11** (0.018 g, 0.044 mmol, 49% yield). ^1^H NMR (300 MHz, CDCl_3_) δ ppm 0.79 (t, *J* = 7.16 Hz, 3H), 1.06–1.33 (m, 4H), 1.83–1.97 (m, 1H), 2.14–2.43 (m, 3H), 3.18–3.31 (m, 1H), 3.36–3.51 (m, 1H), 3.85 (dt, *J* = 18.84, 2.64 Hz, 1H), 4.26–4.39 (m, 3H), 4.90 (t, *J* = 5.27 Hz, 1H), 7.11–7.36 (m, 10H); MS (ESI) *m*/*z*: calcd for C_25_H_28_N_2_O_3_ 404.50, found 405.6 [M + H]^+^. HPLC (220 nm) *t*_R_ = 16.05 min. 

#### 4.4.12. N-3-Dibenzyl-1-oxo-3-phenyl-1H,3H,4H,5H,6H,7H-furo[3,4-c]pyridine-5-carboxamide (**12**)

Amine **55** was reacted with benzyl isocyanate according to procedure D to yield compound **12** (0.036 g, 0.082 mmol, 50% yield). ^1^H NMR (300 MHz, CDCl_3_) δ ppm 1.93–2.08 (m, 1H), 2.11–2.23 (m, 1H), 3.11–3.35 (m, 2H), 3.39–3.49 (m, 1H), 3.52–3.62 (m, 1H), 3.97–4.08 (m, 1H), 4.42 (dd, *J* = 5.27, 2.64 Hz, 2H), 4.59 (dt, *J* = 19.12, 2.12 Hz, 1H), 4.90 (t, *J* = 5.46 Hz, 1H), 7.15–7.47 (m, 15H); MS (ESI) *m*/*z*: calcd for C_28_H_26_N_2_O_3_ 438.52, found 439.6 [M + H]^+^. HPLC (220 nm) *t*_R_ = 15.66 min. 

#### 4.4.13. N-Benzyl-3-(2,2-dimethylpropyl)-1-oxo-3-phenyl-1H,3H,4H,5H,6H,7H-furo[3,4-c]pyridine-5-carboxamide (**13**)

Amine **59** was reacted with benzyl isocyanate according to procedure D to yield compound **13** (0.041 g, 0.098 mmol, 65% yield). ^1^H NMR (300 MHz, CDCl_3_) δ ppm 0.87 (s, 9H), 1.88 (d, *J* = 14.88 Hz, 1H), 2.28–2.43 (m, 2H), 2.38 (d, *J* = 14.88 Hz, 1H), 3.16–3.28 (m, 1H), 3.53 (s, 1H), 3.96 (dt, *J* = 19.03, 2.50 Hz, 1H), 4.42 (d, *J* = 5.65 Hz, 2H), 4.59 (s, 1H), 4.88–4.99 (m, 1H), 7.20–7.41 (m, 10H); MS (ESI) *m*/*z*: calcd for C_26_H_30_N_2_O_3_ 418.53, found 419.4 [M + H]^+^. HPLC (220 nm) *t*_R_ = 16.43 min. 

#### 4.4.14. N-Benzyl-3-methyl-1-oxo-3-phenyl-1H,3H,4H,5H,6H,7H-furo[3,4-c]pyridine-5-carboxamide (**15**)

Amine **62** was reacted with benzyl isocyanate according to procedure D to yield compound **15** (0.028 g, 0.077 mmol, 39% yield). ^1^H NMR (300 MHz, CDCl_3_) δ ppm 1.88 (s, 3H), 2.40 (dt, *J* = 5.18, 2.87 Hz, 2H), 3.35 (dt, *J* = 13.56, 6.03 Hz, 1H), 3.55 (dt, *J* = 13.56, 5.27 Hz, 1H), 3.85 (dt, *J* = 19.12, 2.50 Hz, 1H), 4.34 (t, *J* = 2.26 Hz, 1H), 4.38–4.45 (m, 2H), 4.97 (t, *J* = 5.27 Hz, 1H), 7.22–7.41 (m, 10H); MS (ESI) *m*/*z*: calcd for C_22_H_22_N_2_O_3_ 362.42, found 363.4 [M + H]^+^. HPLC (220 nm) *t*_R_ = 14.12 min. 

#### 4.4.15. N-Benzyl-3,3-bis(2-methylpropyl)-1-oxo-1H,3H,4H,5H,6H,7H-furo[3,4-c]pyridine-5-carboxamide (**16**)

Amine **65** was reacted with benzyl isocyanate according to procedure D to yield compound **16** (0.042 g, 0.11 mmol, 36% yield). ^1^H NMR (300 MHz, CDCl_3_) δ ppm 0.75–0.93 (m, 12H), 1.39–1.60 (m, 4H), 1.77–1.92 (m, 2H), 2.28–2.45 (m, 2H), 3.36–3.55 (m, 2H), 4.03–4.18 (m, 2H), 4.34–4.48 (m, 2H), 4.85–5.03 (m, 1H), 7.17–7.39 (m, 5H); MS (ESI) *m*/*z*: calcd for C_23_H_32_N_2_O_3_ 384.51, found 385.4 [M + H]^+^. HPLC (220 nm) *t*_R_ = 16.06 min. 

#### 4.4.16. N-Benzyl-3-[2-(dimethylamino)ethyl]-1-oxo-3-phenyl-1H,3H,4H,5H,6H,7H-furo[3,4-c]pyridine-5-carboxamide (**17**)

Amine **68** was reacted with benzyl isocyanate according to procedure D to yield compound **17** (0.015 g, 0.036 mmol, 28% yield). ^1^H NMR (300 MHz, CDCl_3_) δ ppm 2.15–2.19 (m, 6H), 2.20–2.53 (m, 6H), 3.23–3.38 (m, 1H), 3.59 (d, *J* = 13.56 Hz, 1H), 3.86–3.98 (m, 1H), 4.38–4.44 (m, 2H), 4.49 (t, *J* = 2.07 Hz, 1H), 4.98 (br. s, 1H), 7.20–7.44 (m, 10H); MS (ESI) *m*/*z*: calcd for C_25_H_29_N_3_O_3_ 419.52, found 420.6 [M + H]^+^. HPLC (220 nm) *t*_R_ = 11.95 min. 

#### 4.4.17. N-Benzyl-3-(hydroxymethyl)-1-oxo-3-phenyl-1H,3H,4H,5H,6H,7H-furo[3,4-c]pyridine-5-carboxamide (**18**)

Amine **72** was reacted with benzyl isocyanate according to procedure D to yield compound **18** (0.101 g, 0.27 mmol, 51% yield). ^1^H NMR (300 MHz, CDCl_3_) δ ppm 2.18–2.47 (m, 2H), 3.19–3.31 (m, 1H), 3.63 (dt, *J* = 13.75, 4.99 Hz, 1H), 3.94–4.07 (m, 3H), 4.07–4.18 (m, 1H), 4.35 (d, *J* = 5.65 Hz, 2H), 4.47 (d, *J* = 18.84 Hz, 1H), 5.50 (t, *J* = 5.65 Hz, 1H), 7.15–7.25 (m, 4H), 7.27–7.42 (m, 6H); MS (ESI) *m*/*z*: calcd for C_22_H_22_N_2_O_4_ 378.42, found 379.4 [M + H]^+^. HPLC (220 nm) *t*_R_ = 12.93 min. 

#### 4.4.18. [5-(Benzylcarbamoyl)-1-oxo-3-phenyl-1H,3H,4H,5H,6H,7H-furo[3,4-c]pyridin-3-yl]methyl Acetate (**19**)

Compound **18** (0.05 g, 0.13 mmol, 1 equiv.) was combined with triethylamine (0.016 mL, 0.16 mmol, 1.2 equiv.) and cooled to 0 °C. Acetyl chloride (0.13 g, 0.16 mmol, 1.2 equiv.) was then added slowly and the reaction was allowed to warm to room temperature and stir for 1 h. The reaction was quenched with saturated sodium bicarbonate and extracted with EtOAc. The organic layer was dried with sodium sulfate and concentrated under reduced pressure. The residue was purified by column chromatography to yield compound **19** (0.031 g, 0.073 mmol, 57% yield). ^1^H NMR (300 MHz, CDCl_3_) δ ppm 2.00 (s, 3H), 2.42 (tt, *J* = 4.85, 2.68 Hz, 2H), 3.33 (ddd, *J* = 13.66, 6.50, 5.09 Hz, 1H), 3.58–3.69 (m, 1H), 4.01 (dt, *J* = 18.84, 2.64 Hz, 1H), 4.35–4.45 (m, 3H), 4.50 (d, *J* = 12.06, 1H), 4.94 (t, *J* = 5.46 Hz, 1H), 7.23–7.45 (m, 10H); MS (ESI) *m*/*z*: calcd for C_24_H_24_N_2_O_5_ 420.46, found 421.4 [M + H]^+^. HPLC (220 nm) *t*_R_ = 14.21 min.

#### 4.4.19. N-Benzyl-3-(3-hydroxyphenyl)-3-methyl-1-oxo-1H,3H,4H,5H,6H,7H-furo[3,4-c]pyridine-5-carboxamide (**20**)

Amine **73** was reacted with benzyl isocyanate according to procedure D to yield compound **20** (68 mg, 0.18 mmol, 58% yield). ^1^H NMR (300 MHz, CDCl_3_) δ ppm 1.78–1.88 (m, 3H), 2.31–2.45 (m, 2H), 3.28–3.58 (m, 2H), 3.86–4.00 (m, 1H), 4.26–4.51 (m, 3H), 5.00 (t, *J* = 5.46 Hz, 1H), 6.73–6.88 (m, 3H), 7.04–7.44 (m, 7H); MS (ESI) *m*/*z*: calcd for C_22_H_22_N_2_O_4_ 378.42, found 379.4 [M + H]^+^. HPLC (280 nm) *t*_R_ = 12.88 min.

#### 4.4.20. N-Benzyl-3-[3-(benzyloxy)phenyl]-3-methyl-1-oxo-1H,3H,4H,5H,6H,7H-furo[3,4-c]pyridine-5-carboxamide (**21**)

Amine **77** was reacted with benzyl isocyanate according to procedure D to yield compound **21** (43 mg, 0.09 mmol, 60% yield). ^1^H NMR (300 MHz, CDCl_3_) δ ppm 1.79–1.89 (m, 3H), 2.28–2.45 (m, 2H), 3.21–3.38 (m, 1H), 3.55 (dt, *J* = 13.56, 5.09 Hz, 1H), 3.72–3.88 (m, 1H), 4.27–4.35 (m, 1H), 4.37–4.43 (m, 2H), 4.95 (t, *J* = 5.46 Hz, 1H), 5.04 (s, 2H), 6.83–6.96 (m, 3H), 7.22–7.45 (m, 10H), 8.26–8.28 (m, 1H); MS (ESI) *m*/*z*: calcd for C_29_H_28_N_2_O_4_ 468.54, found 469.6 [M + H]^+^. HPLC (280 nm) *t*_R_ = 16.36 min.

#### 4.4.21. N-Benzyl-3-(2-methylpropyl)-3-(naphthalen-2-yl)-1-oxo-1H,3H,4H,5H,6H,7H-furo[3,4-c]pyridine-5-carboxamide (**22**)

Amine **82** was reacted with benzyl isocyanate according to procedure D to yield compound **22** (77 mg, 0.17 mmol, 89% yield). ^1^H NMR (300 MHz, CDCl_3_) δ ppm 0.88 (d, *J* = 6.78 Hz, 3H), 0.98 (d, *J* = 6.40 Hz, 3H), 1.61–1.78 (m, 1H), 1.81–1.96 (m, 1H), 2.30–2.53 (m, 3H), 3.16–3.32 (m, 1H), 3.55 (dt, *J* = 13.85, 4.94 Hz, 1H), 3.94 (dt, *J* = 19.21, 2.64 Hz, 1H), 4.33–4.49 (m, 2H), 4.60 (d, *J* = 18.84, 1H), 4.80 (t, *J* = 5.46 Hz, 1H), 7.20–7.40 (m, 5H), 7.45–7.58 (m, 2H), 7.79–7.92 (m, 4H); MS (ESI) *m*/*z*: calcd for C_29_H_30_N_2_O_3_ 454.56, found 455.6 [M + H]^+^. HPLC (280 nm) t_R_ = 17.21 min.

#### 4.4.22. N-Benzyl-3-(2-hydroxyphenyl)-3-(2-methylpropyl)-1-oxo-1H,3H,4H,5H,6H,7H-furo[3,4-c]pyridine-5-carboxamide (**23**) 

Amine **88** was reacted with benzyl isocyanate according to procedure D to yield compound **23** (0.02 g, 0.05 mmol, 28% yield over two steps). ^1^H NMR (300 MHz, CDCl_3_) δ ppm 0.75–0.99 (m, 6H), 1.53–1.79 (m, 2H), 2.22–2.56 (m, 3H), 3.30 (dt, *J* = 13.47, 5.32 Hz, 1H), 3.42–3.61 (m, 1H), 3.94 (d, *J* = 19.21 Hz, 1H), 4.26–4.54 (m, 3H), 5.06 (t, *J* = 5.46 Hz, 1H), 5.95 (t, *J* = 6.03 Hz, 1H), 7.08–7.57 (m, 9H); MS (ESI) *m*/*z*: calcd for C_25_H_28_N_2_O_4_ 420.50, found 421.4 [M + H]^+^. 

#### 4.4.23. N-Benzyl-3-[2-(benzyloxy)phenyl]-3-(2-methylpropyl)-1-oxo-1H,3H,4H,5H,6H,7H-furo[3,4-c]pyridine-5-carboxamide (**24**)

Amine **87** was reacted with benzyl isocyanate according to procedure D to yield compound **24** (0.021 g, 0.04 mmol, 44% yield). ^1^H NMR (300 MHz, CDCl_3_) δ ppm 0.85 (dd, *J* = 15.07, 6.40 Hz, 6H), 1.48–1.69 (m, 2H), 2.13–2.41 (m, 2H), 2.53–2.69 (m, 1H), 3.17 (ddd, *J* = 13.09, 8.01, 4.90 Hz, 1H), 3.69 (dt, *J* = 13.37, 4.99 Hz, 1H), 3.92–4.02 (m, 1H), 4.17 (dd, *J* = 12.81, 6.40 Hz, 1H), 4.33 (d, *J* = 5.27 Hz, 2H), 4.99–5.11 (m, 2H), 6.96–7.08 (m, 2H), 7.20–7.47 (m, 12H), 7.59 (dd, *J* = 8.10, 1.70 Hz, 1H); MS (ESI) *m*/*z*: calcd for C_32_H_34_N_2_O_4_ 510.62, found 511.6 [M + H]^+^. HPLC (280 nm) *t*_R_ = 18.02 min.

#### 4.4.24. N-Benzyl-3-(3-chlorophenyl)-3-(2-methylpropyl)-1-oxo-1H,3H,4H,5H,6H,7H-furo[3,4-c]pyridine-5-carboxamide (**25**)

Amine **91** was reacted with benzyl isocyanate according to procedure D to yield compound **24** (0.105 g, 0.23 mmol, quantitative yield). ^1^H NMR (300 MHz, CDCl_3_) δ ppm 0.85 (d, *J* = 6.78 Hz, 3H), 0.94 (d, *J* = 6.40 Hz, 3H), 1.54–1.69 (m, 1H), 1.71–1.84 (m, 1H), 2.26 (dd, *J* = 14.69, 4.90 Hz, 2H), 2.30–2.39 (m, 1H), 3.24 (ddd, *J* = 13.37, 7.72, 5.27 Hz, 1H), 3.57 (dt, *J* = 13.56, 5.09 Hz, 1H), 3.90 (dt, *J* = 19.21, 2.64 Hz, 1H), 4.39 (d, *J* = 5.27 Hz, 2H), 4.43–4.54 (m, 1H), 5.22 (t, *J* = 5.46 Hz, 1H), 7.14–7.37 (m, 9H); MS (ESI) *m*/*z*: calcd for C_25_H_27_ClN_2_O_3_ 438.95, found 439.6 [M + H]^+^. HPLC (220 nm) *t*_R_ = 16.85 min.

#### 4.4.25. N-Butyl-3-(2-methylpropyl)-1-oxo-3-phenyl-1H,3H,4H,5H,6H,7H-furo[3,4-c]pyridine-5-carboxamide (**26**)

Amine **33** was reacted with benzyl isocyanate according to procedure D to yield compound **26** (0.054 g, 0.15 mmol, quantitative yield). ^1^H NMR (300 MHz, CDCl_3_) δ ppm 0.79–1.01 (m, 9H), 1.18–1.42 (m, 3H), 1.42–1.56 (m, 2H), 1.56–1.73 (m, 1H), 1.75–1.88 (m, 1H), 2.26–2.51 (m, 2H), 3.15–3.35 (m, 2H), 3.53 (dt, *J* = 13.75, 4.99 Hz, 1H), 3.82–3.96 (m, 1H), 4.41–4.55 (m, 1H), 4.64 (t, *J* = 5.27 Hz, 1H), 7.23–7.49 (m, 5H); MS (ESI) *m*/*z*: calcd for C_22_H_30_N_2_O_3_370.49, found 371.4 [M + H]^+^. HPLC (220 nm) *t*_R_ = 15.88 min.

#### 4.4.26. 3-(2-Methylpropyl)-1-oxo-3-phenyl-N-[2-(piperidin-1-yl)Ethyl]-1H,3H,4H,5H,6H,7H-furo[3,4-c]pyridine-5-carboxamide (**27**)

Triphosgene (0.023 g, 0.09 mmol, 1 equiv.) was added to amine **33** (0.07 g, 0.26 mmol, 2.9 equiv.) in THF at 0 °C, and the reaction was allowed to stir for 10 min. Triethylamine (0.053 g, 0.52 mmol, 5.8 equiv.) was then added slowly and the reaction was stirred for 15 min. 1-(2-aminoethyl)piperidine (0.033 g, 0.26 mmol, 2.9 equiv.) was then added slowly and the reaction was allowed to adjust to room temperature over 30 min. The reaction was quenched with saturated sodium bicarbonate and extracted with EtOAc. The organic layer was dried with sodium sulfate and concentrated under reduced pressure. The residue was purified by column chromatography to yield compound **27** (0.061 g, 0.14 mmol, 55% yield). ^1^H NMR (300 MHz, CDCl_3_) δ ppm 0.86 (d, *J* = 6.78 Hz, 3H), 0.95 (d, *J* = 6.78 Hz, 3H), 1.40–1.72 (m, 7H), 1.81 (dd, *J* = 14.51,7.35 Hz, 1H), 2.25–2.55 (m, 9H), 3.25–3.43 (m, 3H), 3.55 (dt, *J* = 13.56, 5.27 Hz, 1H), 3.91 (dt, *J* = 19.12, 2.50 Hz, 1H), 4.42–4.56 (m, 1H), 5.70 (t, *J* = 4.33 Hz, 1H), 7.24–7.49 (m, 5H); MS (ESI) *m*/*z*: calcd for C_25_H_35_N_3_O_3_ 425.56, found 426.6 [M + H]^+^. HPLC (220 nm) *t*_R_ = 13.43 min.

#### 4.4.27. 3-(2-Methylpropyl)-1-oxo-3-phenyl-N-[2-(piperidin-1-yl)ethyl]-1H,3H,4H,5H,6H,7H-furo[3,4-c]pyridine-5-carbothioamide (**28**)

Amine **33** was reacted with 2-piperidinoethyl isothiocyanate according to procedure D to yield compound **28** (0.019 g, 0.043 mmol, 29% yield). ^1^H NMR (300 MHz, CDCl_3_) δ ppm 0.81–0.91 (m, 3H), 0.97 (m, 3H), 1.41–1.63 (m, 5H), 1.63–1.75 (m, 1H), 1.84 (dd, *J* = 14.69, 7.16 Hz, 1H), 2.27–2.62 (m, 10H), 3.56–3.75 (m, 3H), 3.90 (dt, *J* = 13.85, 4.95 Hz, 1H), 4.27 (dt, *J* = 18.93, 2.40 Hz, 1H), 5.18 (d, *J* = 18.84 Hz, 1H), 7.02 (br s, 1H), 7.26–7.44 (m, 5H); MS (ESI) *m*/*z*: calcd for C_25_H_35_N_3_O_2_S 441.63, found 442.6 [M + H]^+^. HPLC (220 nm) *t*_R_ = 14.19 min.

#### 4.4.28. N-Benzyl-3-(2-methylpropyl)-1-oxo-3-phenyl-hexahydro-1H-[1,3]oxazolo[3,4-a]piperazine-5-carboxamide (**29**)

Amine **95** was reacted with 2-piperidinoethyl isothiocyanate according to procedure D to yield compound **29** (0.110 g, 0.27 mmol, quantitative yield). ^1^H NMR (300 MHz, CDCl_3_) δ ppm 0.70 (d, *J* = 6.78 Hz, 3H), 0.91 (d, *J* = 6.78 Hz, 3H), 1.59 (dt, *J* = 12.62, 6.50 Hz, 1H), 1.80–1.97 (m, 1H), 1.99–2.13 (m, 1H), 2.74 (td, *J* = 12.81, 3.58 Hz, 1H), 3.02 (td, *J* = 12.72, 3.58 Hz, 1H), 3.51–3.92 (m, 4H), 4.35 (d, *J* = 5.46 Hz, 2H), 5.01 (t, *J* = 5.46 Hz, 1H), 6.80–7.57 (m, 5H); MS (ESI) *m*/*z*: calcd for C_24_H_29_N_3_O_3_ 407.51, found 408.4 [M + H]^+^. HPLC (220 nm) *t*_R_ = 3.49 min.

#### 4.4.29. 3-[Hydroxy(diphenyl)methyl]-N,N-bis(1-methylethyl)pyridine-4-carboxamide (**34**)

The alcohol intermediate was synthesized from N,N-diisopropylisonicotinamide and benzophenone according to procedure A to yield the desired compound (0.617 g, 1.59 mmol, 54% yield). ^1^H NMR (300 MHz, CDCl_3_) δ ppm 0.80 (d, *J* = 6.40 Hz, 3H), 1.06 (d, *J* = 6.78 Hz, 3H), 1.18 (d, *J* = 6.40 Hz, 3H), 1.33–1.49 (m, 3H), 1.41 (d *J* = 6.78 Hz, 3H), 3.19–3.36 (m, 1H), 3.60 (spt, *J* = 6.59 Hz, 1H), 6.65 (d, *J* = 11.30 Hz, 1H), 7.22–7.42 (m, 13H), 8.13 (d, *J* = 0.75, 1H), 8.55 (d, *J* = 4.90 Hz, 1H). 

#### 4.4.30. 3,3-Diphenylfuro[3,4-c]pyridin-1(3H)-one (**35**)

The pyridine intermediate was synthesized from compound **34** using procedure B to yield the desired compound (0.368 g, 1.29 mmol, 82% yield). ^1^H NMR (300 MHz, CDCl_3_) δ ppm 7.29–7.40 (m, 10H), 7.77–7.88 (m, 1H), 8.89 (d, *J* = 4.90 Hz, 1H), 9.04 (d, *J* = 0.75 Hz, 1H).

#### 4.4.31. 3,3-Diphenyl-4,5,6,7-tetrahydrofuro[3,4-c]pyridin-1(3H)-one (**36**)

The reduced analog was synthesized from compound **35** using procedure C to yield the desired compound (0.260 g, 0.89 mmol, 73% yield). ^1^H NMR (300 MHz, CDCl_3_) δ ppm 2.37 (tt, *J* = 5.51, 2.59 Hz, 2H), 3.01 (t, *J* = 5.65 Hz, 2H), 3.63 (t, *J* = 2.45 Hz, 2H), 7.19–7.29 (m, 10H), 7.32–7.37 (m, 10H). 

#### 4.4.32. (3,7)-3,3-Diphenylhexahydrofuro[3,4-c]pyridin-1(3H)-one (**37**)

Intermediate **36** (0.26 g, 0.89 mmol, 1 equiv.) and NiOAc• 4H_2_O (0.33 g, 1.34 mmol, 1.5 equiv.) were suspended in methanol (5 mL, 0.2 M). NaBH_4_ (0.25 g, 6.7 mmol, 7.5 equiv.) was then added. The reaction was stirred for 2 h. The reaction was quenched with H_2_O and extracted with EtOAc. The organic layer was dried with sodium sulfate and concentrated under reduced pressure to yield the reduced *cis*-product (0.086 g, 0.29 mmol, 33% yield). ^1^H NMR (300 MHz, CDCl_3_) δ ppm 1.71–1.87 (m, 1H), 2.06–2.21 (m, 2H), 2.45 (td, *J* = 12.43, 3.01 Hz, 1H), 2.70 (dd, *J* = 12.43, 5.65 Hz, 1H), 2.80–2.97 (m, 2H), 3.44 (dt, *J* = 11.68, 5.84 Hz, 1H), 7.13–7.43 (m, 8H), 7.51–7.58 (m, 2H). 

#### 4.4.33. (3,7)-3,3-Diphenylhexahydrofuro[3,4-c]pyridin-1(3H)-one and (3aS,7aR)-3,3-Diphenylhexahydrofuro[3,4-c]pyridin-1(3H)-one Mixture (**38**)

*Cis* amine 37 (0.06 g, 0.20 mmol, 1 equiv.) was dissolved in THF (5 mL, 0.04 M) at 0 °C. NaH (0.02 g, 0.51 mmol, 2.6 equiv.) was added and the reaction was stirred for 30 min. The resulting mixture of *cis* and *trans* compounds was inseparable at this stage and was carried forward without further purification. 

#### 4.4.34. 1,1-Diphenylfuro[3,4-c]pyridin-3(1H)-one (**39**)

Phenyl magnesium bromide (15 mL (1 M), 15 mmol, 2.2 equiv.) was added to pyridine-3,4-dicarboxylic anhydride (1.0 g, 6.7 mmol, 1 equiv.) in THF (50 mL, 0.13 M) at −78 °C. The reaction was allowed to warm to room temperature. The reaction was quenched with water and extracted with CH_2_Cl_2_. The organic layer was dried with sodium sulfate and concentrated. The resulting material was carried forward without further purification.

#### 4.4.35. 1,1-Diphenyl-4,5,6,7-tetrahydrofuro[3,4-c]pyridin-3(1H)-one (**40**)

Pyridine **39** was reduced using procedure C to yield compound **40** (0.87 g, 2.98 mmol, 86% yield). ^1^H NMR (300 MHz, CDCl_3_) δ ppm 2.36 (tt, *J* = 5.46, 2.45 Hz, 2H), 3.00 (t, *J* = 5.46 Hz, 2H), 3.63 (t, *J* = 2.45 Hz, 2H), 7.20–7.27 (m, 4H), 7.29–7.40 (m, 6H). 

#### 4.4.36. 3-(9-Hydroxy-9H-fluoren-9-yl)-N,N-bis(1-methylethyl)pyridine-4-carboxamide (**41**)

The alcohol intermediate **41** was synthesized from N,N-diisopropylisonicotinamide and 9-fluorenone according to procedure A to yield the desired compound (0.79 g, 1.83 mmol, 38% yield). ^1^H NMR (300 MHz, CDCl_3_) δ ppm 1.15–1.23 (m, 3H), 1.32 (d, *J* = 6.40 Hz, 3H), 1.54 (d, *J* = 6.78 Hz, 3H), 1.61 (d, *J* = 6.78 Hz, 3H), 3.48–3.53 (m, 1H), 3.53–3.64 (m, 1H), 3.81–3.95 (m, 1H), 7.03–7.07 (m, 1H), 7.15–7.36 (m, 4H), 7.42 (td, *J* = 7.54, 1.13 Hz, 1H), 7.60–7.72 (m, 3H), 7.78–7.85 (m, 1H), 8.34 (d, *J* = 4.90 Hz, 1H). 

#### 4.4.37. 1′H-Spiro[fluorene-9,3′-furo[3,4-c]pyridin]-1′-one (**42**)

The pyridine intermediate was synthesized from compound **41** using procedure B to yield the desired compound which was carried forward without further purification. 

#### 4.4.38. 1′H-Spiro[fluorene-9,3′-furo[3,4-c]pyridin]-1′-one (**43**)

The reduced analog was synthesized from pyridine **42** using procedure C to yield the desired compound (0.28 g, 0.97 mmol, 77% yield). ^1^H NMR (300 MHz, CDCl_3_) δ ppm 2.49–2.53 (m, 3H), 3.05–3.05 (m, 4H), 7.21 (d, *J* = 7.50 Hz, 2H), 7.29–7.31 (m, 2H), 7.46 (t, *J* = 6.30 Hz, 2H), 7.67 (d, *J* = 7.50 Hz, 2H). 

#### 4.4.39. 3-(5-Hydroxy-10,11-dihydro-5H-dibenzo[a,d][7]annulen-5-yl)-N,N-bis(1-methYlethyl)pyridine-4-carboxamide (**44**)

The alcohol intermediate was synthesized from N,N-diisopropylisonicotinamide and dibenzosuberone according to procedure A to yield the desired compound (0.66 g, 1.44 mmol, 30% yield). ^1^H NMR (300 MHz, CDCl_3_) δ ppm 0.55 (d, *J* = 6.40 Hz, 3H), 0.98–1.05 (m, 3H), 1.15 (d, *J* = 6.78 Hz, 3H), 1.36 (d, *J* = 6.78 Hz, 3H), 2.43–2.56 (m, 1H), 2.58–2.78 (m, 1H), 2.93–3.25 (m, 4H(, 3.44 (dt, *J* = 13.19, 6.59 Hz, 1H), 6.90 (s, 1H), 6.97–7.25 (m, 8H), 7.94 (dd, *J* = 7.54, 1.51 Hz, 1H), 8.04–8.14 (m, 1H), 8.35 (s, 1H), 8.56 (d, *J* = 4.90 Hz, 1H). 

#### 4.4.40. 10,11-Dihydro-1′H-spiro[dibenzo[a,d][7]annulene-5,3′-furo[3,4-c]pyridin]-1′-one (**45**)

The pyridine intermediate was synthesized from compound **44** using procedure B to yield the desired compound (0.147 g, 0.47 mmol, 33% yield). ^1^H NMR (300 MHz, CDCl_3_) δ ppm 3.04–3.20 (m, 2H), 3.76–3.95 (m, 2H), 6.84–7.12 (m, 8H), 7.90 (d, *J* = 4.52 Hz, 1H), 8.89–9.03 (m, 1H), 9.21 (s, 1H). 

#### 4.4.41. 4′,5′,6′,7′,10,11-Hexahydro-1′H-spiro[dibenzo[a,d][7]annulene-5,3′-furo[3,4-c]pyridin]-1′-one (**46**)

The reduced analog was synthesized from pyridine **45** using procedure C to yield the desired compound (0.053 g, 0.17 mmol, 36% yield). ^1^H NMR (300 MHz, CDCl_3_) δ ppm 1.70 (br s, 1H), 2.27–2.37 (m, 2H), 2.90 (t, *J* = 5.65 Hz, 2H), 2.96–3.19 (m, 4H), 3.44 (t, *J* = 2.45 Hz, 2H), 7.03–7.31 (m, 8H). 

#### 4.4.42. 3-(5-Hydroxy-6,7,8,9-tetrahydro-5H-benzo[7]annulen-5-yl)-N,N-bis(1-methylethyl)pyridine-4-carboxamide (**47**)

The alcohol intermediate was synthesized from N,N-diisopropylisonicotinamide and 1-benzosuberone according to procedure A to yield the desired compound, which was carried forward without further purification.

#### 4.4.43. 6,7,8,9-Tetrahydro-1′H-spiro[benzo[7]annulene-5,3′-furo[3,4-c]pyridin]-1′-one (**48**)

The pyridine intermediate was synthesized from compound **47** using procedure B to yield the desired compound (0.400 g, 1.51 mmol, 62% yield). ^1^H NMR (300 MHz, CDCl_3_) δ ppm 1.68 (dtdd, *J* = 13.40, 11.48, 3.58, 1.88 Hz, 1H), 1.87–2.36 (m, 4H), 2.34–2.48 (m, 1H), 2.90 (dd, *J* = 14.32, 6.78 Hz, 1H), 3.47 (ddd, *J* = 14.03, 11.59, 1.88 Hz, 1H), 6.79–6.88 (m, 1H), 6.97–7.07 (m, 1H), 7.18–7.25 (m, 2H), 7.76–7.85 (m, 1H), 8.85–8.92 (m, 1H), 8.96–9.08 (m, 1H). 

#### 4.4.44. 4′,5′,6,6′,7,7′,8,9-Octahydro-1′H-spiro[benzo[7]annulene-5,3′-furo[3,4-c]pyridin]-1′-one (**49**)

The reduced analog was synthesized from pyridine **48** using procedure C to yield the desired compound (0.28 g, 1.04 mmol, 67% yield). ^1^H NMR (300 MHz, CDCl_3_) δ ppm 1.56–2.23 (m, 5H), 2.23–2.44 (m, 2H), 2.74–3.18 (m, 4H), 3.50–3.75 (m, 2H), 6.92–7.02 (m, 1H), 7.06–7.25 (m, 3H). 

#### 4.4.45. 3-(1-Hydroxy-1-phenylpentyl)-N,N-bis(1-methylethyl)pyridine-4-carboxamide (**50**)

The alcohol intermediate was synthesized from N,N-diisopropylisonicotinamide and valerophenone according to procedure A to yield the desired compound (1.2 g, 2.90 mmol, 30% yield). ^1^H NMR (300 MHz, CDCl_3_) δ ppm 0.38 (d, J-6.78 Hz, 3H), 0.71–0.93 (m, 4H), 1.06 (d, *J* = 6.40 Hz, 6H), 1.35–1.44 (m, 3H), 1.50–1.58 (m, 3H), 2.02–2.16 (m, 1H), 2.29–2.43 (m, 1H), 3.22 (dquin, *J* = 9.04, 6.69 Hz, 1H), 3.54 (dt, *J* = 13.66, 6.92 Hz, 1H), 3.76 (quin, *J* = 6.59 Hz, 1H), 7.03 (d, *J* = 4.90 Hz, 1H), 7.21–7.32 (m, 5H), 8.51–8.59 (m, 1H), 9.00 (s, 1H). 

#### 4.4.46. 3-Butyl-3-phenylfuro[3,4-c]pyridin-1(3H)-one (**51**)

The pyridine intermediate was synthesized from alcohol **50** using procedure B to yield the desired compound (0.83 g, 3.10 mmol, quantitative yield). ^1^H NMR (300 MHz, CDCl_3_) δ ppm 0.70–0.79 (m, 3H), 0.94–1.10 (m, 1H), 1.15–1.32 (m, 3H), 2.13–2.28 (m, 1H), 2.36–2.54 (m, 1H), 7.21–7.37 (m, 3H), 7.44–7.54 (m, 2H), 7.68–7.76 (m, 1H), 8.73–8.82 (m, 1H), 8.99–9.03 (s, 1H). 

#### 4.4.47. 3-Butyl-3-phenyl-4,5,6,7-tetrahydrofuro[3,4-c]pyridin-1(3H)-one (**52**)

The reduced analog was synthesized from pyridine **51** using procedure C to yield the desired compound (0.48 g, 1.77 mmol, 57% yield). ^1^H NMR (300 MHz, CDCl_3_) δ ppm 0.82–0.93 (m, 3H), 1.17–1.42 (m, 5H), 1.63 (br s, 1H), 1.87–2.01 (m, 1H), 2.15–2.41 (m, 3H), 2.82–3.05 (m, 2H), 3.37–3.50 (m, 1H), 3.56–3.69 (m, 1H), 7.25–7.42 (m, 5H). 

#### 4.4.48. 3-(1-Hydroxy-1,2-diphenylethyl)-N,N-bis(1-methylethyl)pyridine-4-carboxamide (**53**)

The alcohol intermediate was synthesized from N,N-diisopropylisonicotinamide and benzyl phenyl ketone according to procedure A and recrystallized from EtOAc to yield the desired compound (1.73 g, 3.87 mmol, 40% yield). ^1^H NMR (300 MHz, CDCl_3_) δ ppm 0.35 (d, *J* = 6.40 Hz, 3H), 1.01–1.06 (m, 3H), 1.12 (d, J= 6.78 Hz, 3H), 1.32–1.41 (m, 3H), 3.10–3.29 (m, 2H), 3.32–3.43 (m, 1H), 3.73–3.85 (m, 1H), 6.23 (d, *J* = 1.88 Hz, 1H), 6.73–6.81 (m, 1H), 7.00–7.20 (m, 10H), 8.55–8.66 (m, 1H), 9.15 (s, 1H). 

#### 4.4.49. 3-Benzyl-3-phenylfuro[3,4-c]pyridin-1(3H)-one (**54**)

The pyridine intermediate was synthesized from alcohol **53** using procedure B to yield the desired compound (1.19 g, 3.95 mmol, quantitative yield). ^1^H NMR (300 MHz, CDCl_3_) δ ppm 3.69 (q, *J* = 14.07 Hz, 2H), 6.84–6.93 (m, 2H), 7.06–7.15 (m, 3H), 7.33–7.47 (m, 3H), 7.54 (dd, *J* = 4.90, 1.13 Hz, 1H), 7.57–7.64 (m, 2H), 8.74 (d, *J* = 4.90 Hz, 1H), 9.03 (d, *J* = 1.13 Hz, 1H). 

#### 4.4.50. 3-Benzyl-3-phenyl-4,5,6,7-tetrahydrofuro[3,4-c]pyridin-1(3H)-one (**55**)

The reduced analog was synthesized from pyridine **54** using procedure C to yield the desired compound (0.22 g, 0.72 mmol, 18% yield). ^1^H NMR (300 MHz, CDCl_3_) δ ppm 1.49 (br s, 1H), 1.75–2.04 (m, 2H), 2.56–2.78 (m, 2H), 3.23–3.32 (m, 1H), 3.33–3.51 (m, 2H), 3.62–3.75 (m, 1H), 7.03–7.18 (m, 5H), 7.20–7.34 (m, 5H). 

#### 4.4.51. 3,3-Dimethyl-1-phenylbutan-1-one (**56**)

t-Butylacetyl chloride (10 g, 74.3 mmol, 1 equiv.) was added dropwise to AlCl_3_ (14.6 g, 111.45 mmol, 1.5 equiv.) in benzene (100 mL, 0.7 M). The reaction was heated to reflux for 8 h, cooled, and then quenched with water and extracted with EtOAc. The organic layer was dried with sodium sulfate, filtered, and concentrated to yield the desired ketone (13.8 g, 78.29 mmol, quantitative). ^1^H NMR (300 MHz, CDCl_3_) δ ppm 1.07 (s, 9H), 2.86 (s, 2H), 7.43–7.46 (m, 2H), 7.52–7.55 (m, 1H), 7.94–7.95 (m, 2H). 

#### 4.4.52. 3-(1-Hydroxy-3,3-dimethyl-1-phenylbutyl)-N,N-bis(1-methylethyl)pyridine-4-carboxamide (**57**)

The alcohol intermediate was synthesized from N,N-diisopropylisonicotinamide and ketone **56** according to procedure A to yield the desired compound (1.8 g, 4.21 mmol, 43% yield). ^1^H NMR (300 MHz, CDCl_3_) δ ppm 0.40 (d, *J* = 6.59 Hz, 3H), 0.77 (s, 9H), 1.03 (d, *J* = 6.40 Hz, 3H), 1.36 (d, *J* = 6.78 Hz, 3H), 1.42 (d, *J* = 6.78 Hz, 3H), 2.27 (dd, *J* = 13.94, 1.70 Hz, 1H), 2.43–2.56 (m, 1H), 3.04–3.18 (m, 1H), 3.19–3.36 (m, 1H), 5.85 (d, *J* = 1.51 Hz, 1H), 6.99 (d, *J* = 5.09 Hz, 1H), 7.14–7.29 (m, 3H), 7.37–7.43 (m, 2H), 8.51 (d, *J* = 4.90 Hz, 1H), 9.01 (s, 1H).

#### 4.4.53. 3-(2,2-Dimethylpropyl)-3-phenylfuro[3,4-c]pyridin-1(3H)-one (**58**)

The pyridine intermediate was synthesized from alcohol **57** using procedure B to yield the desired compound (1.5 g, 5.33 mmol, quantitative yield). ^1^H NMR (300 MHz, CDCl_3_) δ ppm 0.83 (s, 9H), 2.18 (d, *J* = 15.07 Hz, 1H), 2.65 (d, *J* = 15.07 Hz, 1H), 7.23–7.33 (m, 1H), 7.33–7.45 (m, 2H), 7.55–7.67 (m, 2H), 7.75 (dd, *J* = 5.09, 1.13 Hz, 1H), 8.81 (d, *J* = 5.09 Hz, 1H), 9.16 (d, *J* = 0.94 Hz, 1H).

#### 4.4.54. 3-(2,2-Dimethylpropyl)-3-phenyl-4,5,6,7-tetrahydrofuro[3,4-c]pyridin-1(3H)-one (**59**)

The reduced analog was synthesized from pyridine **58** using procedure C to yield the desired compound (1.21 g, 4.24 mmol, 80% yield). ^1^H NMR (300 MHz, CDCl_3_) δ ppm ^1^H NMR (300 MHz, CHLOROFORM-*d*) d ppm 0.88 (s, 9H), 1.86 (d, *J* = 14.88 Hz, 1H), 2.21–2.35 (m, 2H), 2.32 (d, *J* = 14.88 Hz, 1H), 2.74 (s, 1H), 2.78–2.92 (m, 1H), 3.00 (s, 1H), 3.55 (br. s, 1H), 3.74 (s, 1H), 7.05–7.56 (m, 5H).

#### 4.4.55. 3-(1-Hydroxy-1-phenylethyl)-N,N-bis(1-methylethyl)pyridine-4-carboxamide (**60**)

The alcohol intermediate was synthesized from N,N-diisopropylisonicotinamide and acetophenone according to procedure A to yield the desired compound (2.1 g, 5.66 mmol, 58% yield). ^1^H NMR (300 MHz, CDCl_3_) δ ppm 0.43 (d, *J* = 6.78 Hz, 3H), 1.03–1.11 (m, 3H), 1.15–1.24 (m, 6H), 1.33–1.43 (m, 3H), 3.14–3.37 (m, 2H), 6.30–6.50 (m, 1H), 6.96–7.09 (m, 1H), 7.13–7.38 (m, 5H), 8.52–8.58 (m, 1H), 8.93 (s, 1H). 

#### 4.4.56. 3-Methyl-3-phenylfuro[3,4-c]pyridin-1(3H)-one (**61**)

The pyridine intermediate was synthesized from alcohol **60** using procedure B to yield the desired compound (0.60 g, 2.66 mmol, 82% yield). ^1^H NMR (300 MHz, CDCl_3_) δ ppm 2.07–2.13 (m, 3H), 7.28–7.41 (m, 3H), 7.46–7.51 (m, 2H), 7.79 (dd, *J* = 4.90, 1.13 Hz, 1H), 8.86 (d, *J* = 4.90 Hz, 1H), 8.94–9.00 (m, 1H). 

#### 4.4.57. 3-Methyl-3-phenyl-4,5,6,7-tetrahydrofuro[3,4-c]pyridin-1(3H)-one (**62**)

The reduced analog was synthesized from pyridine **61** using procedure C to yield the desired compound (0.44 g, 1.91 mmol, 72% yield). ^1^H NMR (300 MHz, CDCl_3_) δ ppm 1.75 (br s, 1H), 1.83 (s, 3H), 2.29 (dq, *J* = 5.37, 2.48 Hz, 2H), 2.84–3.03 (m, 2H), 3.26–3.38 (m, 1H), 3.53–3.70 (m, 1H), 7.26–7.39 (m, 5H). 

#### 4.4.58. 3-[1-Hydroxy-3-methyl-1-(2-methylpropyl)butyl]-N,N-bis(1-methylethyl)pyridine-4-carboxamide (**63**)

The alcohol intermediate was synthesized from N,N-diisopropylisonicotinamide and 2,6-dimethyl-4-heptanone according to procedure A to yield the desired compound (2.16 g, 5.49 mmol, 57% yield). ^1^H NMR (300 MHz, CDCl_3_) δ ppm 0.66–0.79 (m, 6H), 0.87–1.01 (m, 6H), 1.13–1.23 (m, 6H), 1.49–1.58 (m, 6H), 1.49–1.58 (m, 6H), 1.71–1.91 (m, 6H), 3.15 (s, 1H), 3.43–3.58 (m, 1H), 3.61–3.76 (m, 1H), 6.95–7.03 (m, 1H), 8.38–8.47 (m, 1H), 8.52 (s, 1H). 

#### 4.4.59. 3,3-bis(2-Methylpropyl)furo[3,4-c]pyridin-1(3H)-one (**64**)

The pyridine intermediate was synthesized from alcohol **63** using procedure B to yield the desired compound which was carried forward without further purification. 

#### 4.4.60. 3,3-bis(2-Methylpropyl)-4,5,6,7-tetrahydrofuro[3,4-c]pyridin-1(3H)-one (**65**)

The reduced analog was synthesized from pyridine **64** using procedure C to yield the desired compound (0.12 g, 0.48 mmol, 60% yield). ^1^H NMR (300 MHz, CDCl_3_) δ ppm 0.89 (dd, *J* = 6.59, 2.83 Hz, 12H), 1.34–1.89 (m, 7H), 2.20–2.35 (m, 2H), 2.90–3.05 (m, 2H), 2.90–3.05 (m, 2H), 3.41–3.54 (m, 2H). 

#### 4.4.61. 3-[3-(Dimethylamino)-1-hydroxy-1-phenylpropyl]-N,N-bis(1-methylethyl)pyridine-4-carboxamide (**66**)

The alcohol intermediate was synthesized from N,N-diisopropylisonicotinamide and 3-(dimethylamino)propiophenone according to procedure A to yield the desired compound (0.450 g, 1.05 mmol, 11% yield). ^1^H NMR (300 MHz, CDCl_3_) δ ppm 0.99–1.10 (m, 3H), 1.19–1.28 (m, 3H), 1.52–1.59 (m, 6H), 2.14–2.23 (m, 6H), 2.57–2.89 (m, 2H), 3.49 (quin, *J* = 6.78 Hz, 1H), 3.55–3.67 (m, 1H), 6.95–7.04 (m, 1H), 7.21–7.29 (m, 1H), 7.32–7.43 (m, 2H), 7.50–7.57 (m, 2H), 8.10–8.15 (m, 1H), 8.34–8.41 (m, 1H). 

#### 4.4.62. 3-[2-(Dimethylamino)ethyl]-3-phenylfuro[3,4-c]pyridin-1(3H)-one (**67**)

The pyridine intermediate was synthesized from alcohol **66** using procedure B to yield the desired compound (0.34 g, 1.20 mmol, quantitative). ^1^H NMR (300 MHz, CDCl_3_) δ ppm 2.07 (s, 6H), 2.15–2.25 (m, 2H), 2.30–2.45 (m, 1H), 2.61–2.78 (m, 1H), 7.18–7.38 (m, 3H), 7.41–7.51 (m, 2H), 7.63–7.72 (m, 1H), 8.76 (d, *J* = 5.27 Hz, 1H), 8.91–9.01 (m, 1H). 

#### 4.4.63. 3-[2-(Dimethylamino)ethyl]-3-phenyl-4,5,6,7-tetrahydrofuro[3,4-c]pyridin-1(3H)-one (**68**)

The reduced analog was synthesized from pyridine **67** using procedure C to yield the desired compound (0.050 g, 0.17 mmol, 25% yield). ^1^H NMR (300 MHz, CDCl_3_) δ ppm 2.06–2.52 (m, 12H), 2.83–3.06 (m, 2H), 3.35–3.48 (m, 1H), 3.62–3.75 (m, 1H), 7.26–7.46 (m, 5H). 

#### 4.4.64. 2-(Methoxymethoxy)-1-phenylethanone (**69**)

Hunig’s base (9.49 g, 73.45 mmol, 1.25 equiv.) was added to 2-hydroxyacetophenone (8 g, 58.76 mmol, 1 equiv.) in THF (50 mL, 1.1 M) at 0 °C. MOMCl (5.91 g, 73.45 mmol, 1.25 equiv.) was slowly added and the reaction was stirred at 0 °C for 2 h, followed by stirring for 2 h at room temperature. The reaction was quenched with saturated sodium bicarbonate and extracted with EtOAc. The organic layer was separated, dried with sodium sulfate, filtered, and concentrated. The material was carried forward without further purification.

#### 4.4.65. 3-[1-Hydroxy-2-(methoxymethoxy)-1-phenylethyl]-N,N-bis(1-methylethyl)pyridine-4-carboxamide (**70**)

The alcohol intermediate was synthesized from N,N-diisopropylisonicotinamide and ketone **69** according to procedure A to yield the desired compound, which was carried forward without further purification. 

#### 4.4.66. 3-(Hydroxymethyl)-3-phenylfuro[3,4-c]pyridin-1(3H)-one (**71**)

The pyridine intermediate was synthesized from alcohol **70** using procedure B to yield the desired compound (0.50 g, 2.07 mmol, 45% yield). ^1^H NMR (300 MHz, DMSO-*d*_6_) δ ppm 4.00–4.17 (m, 1H), 4.20–4.35 (m, 1H), 5.52 (s, 1H), 7.45 (s, 3H), 7.61 (d, *J* = 1.13 Hz, 2H), 7.83–7.93 (m, 1H), 8.89 (s, 1H), 9.30 (d, *J* = 1.13 Hz, 1H). 

#### 4.4.67. 3-(Hydroxymethyl)-3-phenyl-4,5,6,7-tetrahydrofuro[3,4-c]pyridin-1(3H)-one (**72**)

The reduced analog was synthesized from pyridine **71** using procedure C to yield the desired compound (0.128 g, 0.52 mmol, 29% yield). ^1^H NMR (300 MHz, DMSO-*d_6_*) δ ppm 2.00–2.14 (m, 2H), 2.20–2.44 (m, 1H), 2.65–2.85 (m, 2H), 3.46–3.69 (m, 2H), 3.80–3.93 (m, 1H), 3.98–4.09 (m, 1H), 5.26–5.36 (m, 1H), 7.28–7.51 (m, 5H).

#### 4.4.68. 3-(3-Hydroxyphenyl)-3-methyl-4,5,6,7-tetrahydrofuro[3,4-c]pyridin-1(3H)-one (**73**)

Benzyl-amino analog **77** (230 mg, 0.65 mmol, 1 equiv.) was dissolved in EtOAc and Pd/C was added (5 mol%). The mixture was placed on the hydrogenator at 40 psi for 7 h. The mixture was then filtered and concentrated, and the residue was purified by column chromatography to yield the deprotected intermediate X (75 mg, 0.31 mmol, 47% yield). ^1^H NMR (300 MHz, CDCl_3_) δ ppm 1.73–1.84 (m, 3H), 2.19–2.40 (m, 2H), 2.98 (t, *J* = 5.65 Hz, 4H), 3.32–3.46 (m, 1H), 3.54–3.70 (m, 1H), 6.65–6.84 (m, 3H), 7.11–7.30 (m, 1H). 

#### 4.4.69. 3-Benzyloxyacetophenone (**74**)

DMF (50 mL, 0.7 M), benzyl chloride (6.4 mL, 55.1 mmol, 1.5 equiv.) and potassium carbonate (10.1 g, 73.4 mmol, 2 equiv.) were added to 3′-hydroxyacetophenone (5 g, 36.7 mmol, 1 equiv.). The mixture was stirred at 80 °C for 2 h. The reaction was cooled to room temperature, diluted with EtOAc, and washed with water three times. The organic layer was separated, dried with sodium sulfate, and concentrated. The residue was purified by column chromatography to yield the desired ketone (6.6 g, 29.1 mmol, 79% yield). ^1^H NMR (300 MHz, CDCl_3_) δ ppm 2.56 (s, 3H), 5.08 (s, 2H), 7.10–7.21 (m, 1H), 7.27–7.47 (m, 6H), 7.50–7.62 (m, 2H).

#### 4.4.70. 3-(1-Hydroxy-1-phenylethyl)-N,N-bis(1-methylethyl)pyridine-4-carboxamide (**75**)

The alcohol intermediate was synthesized from N,N-diisopropylisonicotinamide and ketone 74 according to procedure A to yield the desired compound as an inseparable mixture with starting material. The impure material was carried forward without further purification.

#### 4.4.71. 3-[3-(Benzyloxy)phenyl]-3-methylfuro[3,4-c]pyridin-1(3H)-one (**76**)

The pyridine intermediate was synthesized from alcohol **75** using procedure B to yield the desired compound (0.70 g, 2.11 mmol, 7% yield over two steps). ^1^H NMR (300 MHz, CDCl_3_) δ ppm 2.06 (s, 3H), 4.97–5.07 (m, 2H), 6.86–7.10 (m, 3H), 7.22–7.47 (m, 6H), 7.67–7.82 (m, 1H), 8.75–9.98 (m, 2H). 

#### 4.4.72. 3-[3-(Benzyloxy)phenyl]-3-methyl-4,5,6,7-tetrahydrofuro[3,4-c]pyridin-1(3H)-one (**77**)

The reduced analog was synthesized from pyridine **76** using procedure C to yield the desired compound (0.35 g, 1.04 mmol, 50% yield). ^1^H NMR (300 MHz, CDCl_3_) δ ppm 1.66 (s, 1H), 1.75–1.86 (m, 3H), 2.19–2.35 (m, 2H), 2.80–3.02 (m, 2H), 3.21–3.35 (m, 1H), 3.45–3.63 (m, 1H), 4.99–5.09 (m, 2H), 6.76–6.98 (m, 4H), 7.22–7.47 (m, 5H). 

#### 4.4.73. 3-Methyl-1-naphthalen-2-ylbutan-1-ol (**78**)

Iso-butyl magnesium chloride (48 mL, 2 M solution, 96 mmol, 1.5 equiv.) was slowly added to 2-napthaldehyde (10 g, 64.0 mmol, 1 equiv.) in THF (50 mL, 1.3 M) at 0 °C. The mixture was stirred for 3 h. The reaction was quenched with water and aqueous ammonium chloride. The reaction was diluted with EtOAc and extracted. Then, 3M HCl was added to break the emulsion. The organic layer was separated, dried with sodium sulfate, filtered, and concentrated. The residue was purified by column chromatography to yield the desired compound (5.8 g, 27.1 mmol, 42% yield). ^1^H NMR (300 MHz, CDCl_3_) δ ppm 0.97–0.99 (m, 6H), 1.55–1.83 (m, 3H), 2.05 (br s, 1H), 4.88–4.92 (m, 1H), 7.43–7.50 (m, 3H), 7.78 (s, 1H), 7.80–7.83 (m, 3H). 

#### 4.4.74. 3-Methyl-1-naphthalen-2-ylbutan-1-one (**79**)

PCC (10.7 g, 49.5 mmol, 2 equiv.) was added to alcohol **78** (5.3 g, 24.7 mmol, 1 equiv.) in CH_2_Cl_2_ (100 mL, 0.25 M). The reaction was stirred, and upon completion, celite was added and the mixture was filtered. The filtrate was concentrated and purified by column chromatography to yield the desired ketone (1.2 g, 5.8 mmol, 23% yield). ^1^H NMR (300 MHz, CDCl_3_) δ ppm 1.03 (d, *J* = 6.5 Hz, 6H), 2.36 (sept, *J* = 6.5 Hz, 1H), 2.96 (d, *J* = 7.0 Hz, 2H), 7.52–7.60 (m, 2H), 7.87 (t, *J* = 8.5 Hz, 2H), 7.96 (d, *J* = 8.0 Hz, 1H), 8.03 (dd, *J* = 8.5, 2.0 Hz, 1H), 8.45 (s, 1H). 

#### 4.4.75. 3-(1-Hydroxy-3-methyl-1-naphthalen-2-ylbutyl)-N,N-bis(1-methylethyl)pyridine-4-carboxamide (**80**)

The alcohol intermediate was synthesized from N,N-diisopropylisonicotinamide and ketone **79** according to procedure A to yield the desired compound (1.37 g, 2.96 mmol, 51% yield). ^1^H NMR (300 MHz, CDCl_3_) δ ppm -0.11 (d, *J* = 6.0 Hz, 3H), 0.55 (d, *J* = 6.0 Hz, 3H), 0.89–1.05 (m, 6H), 1.35 (d, *J* = 7.0 Hz, 2H), 1.56–1.63 (m, 5H), 2.18–2.21 (m, 1H), 2.35–2.42 (m, 2H), 3.01–3.08 (m, 2H), 6.42 (d, *J* = 2.0 Hz, 1H), 7.02 (d, *J* = 5.0 Hz, 1H), 7.20–7.22 (m, 1H), 7.43–4.49 (m, 3H), 7.69 (d, *J* = 8.5 Hz), 7.98 (s, 1H), 8.58 (d, *J* = 5.0 Hz, 1H), 9.08 (s, 1H). 

#### 4.4.76. 3-(2-Methylpropyl)-3-naphthalen-2-ylfuro[3,4-c]pyridin-1(3H)-one (**81**)

The pyridine intermediate was synthesized from alcohol **80** using procedure B to yield the desired compound (0.80 g, 2.52 mmol, 85% yield). ^1^H NMR (300 MHz, CDCl_3_) δ ppm 0.80–0.95 (m, 6H), 1.53–1.78 (m, 1H), 2.15 (dd, *J* = 14.88, 6.97 Hz, 1H), 2.64 (dd, *J* = 14.88, 5.09 Hz, 1H), 7.45–7.56 (m, 2H), 7.65 (dd, *J* = 8.85, 2.07 Hz, 1H), 7.74–7.94 (m, 4H), 7.99 (d, *J* = 1.88 Hz, 1H), 8.84 (d, *J* = 5.27 Hz, 1H), 9.12 (d, *J* = 1.13 Hz, 1H). 

#### 4.4.77. 3-(2-Methylpropyl)-3-naphthalen-2-yl-4,5,6,7-tetrahydrofuro[3,4-c]pyridin-1(3H)-one (**82**)

The reduced analog was synthesized from pyridine **81** using procedure C to yield the desired compound (0.60 g, 0.19 mmol, 8% yield). ^1^H NMR (300 MHz, CDCl_3_) δ ppm 0.88 (d, *J* = 6.78 Hz, 3H), 0.98 (d, *J* = 6.40 Hz, 3H), 1.57 (br s, 1H), 1.71 (dqd, *J* = 13.54, 6.73, 6.73, 6.73, 4.71 Hz, 1H), 1.87 (dd, *J* = 14.32, 7.16 Hz, 1H), 2.17–2.42 (m, 3H), 2.76–2.88 (m, 1H), 2.93–3.05 (m, 1H), 3.40–3.55 (m, 1H), 3.68–3.81 (m, 1H), 7.33 (dd, *J* = 8.67, 2.26 Hz, 1H), 7.45–7.54 (m, 2H), 7.77–7.90 (m, 4H). 

#### 4.4.78. 1-[2-(Benzyloxy)phenyl]-3-methylbutan-1-ol (**83**)

Isobutyl magnesium bromide (16.25 mL (2.0 M in THF), 32.5 mmol, 1.5 equiv.) was added to 2-OBn-benzaldehyde (4.6 g, 21.6 mmol, 1 equiv.) in THF (50 mL, 0.4 M) at −78 °C. The reaction was allowed to warm to room temperature. The reaction was quenched with water and extracted with CH_2_Cl_2_. The organic layer was dried with sodium sulfate and concentrated. The resulting residue was purified by silica gel column chromatography to yield the desired compound (3.15 g, 11.65 mmol, 54% yield). ^1^H NMR (300 MHz, CDCl_3_) δ ppm 0.79–0.96 (m, 6H), 1.46–1.83 (m, 4H), 2.60 (br s, 1H), 4.90–5.07 (m, 2H), 6.78–6.98 (m, 3H), 7.07–7.20 (m, 1H), 7.23–7.44 (m, 5H). 

#### 4.4.79. 1-[2-(Benzyloxy)phenyl]-3-methylbutan-1-one (**84**)

Celite (3 g) was added to alcohol **83** (3.15 g, 11.65 mmol, 1 equiv.) in CH_2_Cl_2_ (50 mL, 0.2 M). PCC (3.76 g, 17.48, 1.5 equiv.) was then added, and the reaction was allowed to stir at room temperature for 2 h. The reaction was filtered and then concentrated. The residue was purified by silica gel column chromatography to yield the desired ketone (3.0 g, 11.18 mmol, 96% yield). ^1^H NMR (300 MHz, CDCl_3_) δ ppm 0.71–0.90 (m, 6H), 2.07–2.30 (m, 1H), 2.75–2.87 (m, 2H), 4.95–5.09 (m, 2H), 6.83–6.99 (m, 3H), 7.18–7.44 (m, 5H), 7.56–7.70 (m, 1H). 

#### 4.4.80. 3-{1-[2-(Benzyloxy)phenyl]-1-hydroxy-3-methylbutyl}-N,N-bis(1-methylethyl)pyridine-4-carboxamide (**85**)

The alcohol intermediate was synthesized from N,N-diisopropylisonicotinamide and ketone **84** according to procedure A to yield the desired compound (1.4 g, 2.70 mmol, 53% yield). ^1^H NMR (300 MHz, CDCl_3_) δ ppm 0.06)d, *J* = 6.78 Hz, 3H), 0.48 (d, *J* = 6.40 Hz, 3H), 0.90–1.03 (m, 6H), 1.20 (d, *J* = 6.78 Hz, 3H), 1.35 (d, *J* = 6.78 Hz, 3H), 1.45–1.63 (m, 2H), 2.24 (dd, *J* = 13.00, 5.09 Hz, 1H), 2.47–2.65 (m, 1H), 2.88–3.22 (m, 1H), 4.69–4.97 (m, 2H), 6.39 (d, *J* = 2.64 Hz, 1H), 6.71–7.02 (m, 4H), 7.11–7.28 (m, 5H), 7.76–7.85 (m, 1H), 8.44–8.56 (m, 1H), 8.92 (s, 1H). 

#### 4.4.81. 3-[2-(Benzyloxy)phenyl]-3-(2-methylpropyl)furo[3,4-c]pyridin-1(3H)-one (**86**)

The pyridine intermediate was synthesized from alcohol **85** using procedure B to yield the desired compound (0.41 g, 1.10 mmol, 41% yield). ^1^H NMR (300 MHz, CDCl_3_) δ ppm 0.69–0.94 (m, 6H), 1.43–1.69 (m, 1H), 1.92 (ddd, *J* = 14.60, 7.63, 4.90 Hz, 1H), 2.69–2.91 (m, 1H), 5.03–5.24 (m, 2H), 6.86–7.08 (m, 2H), 7.19–7.34 (m, 1H), 7.38- 7.50 (m, 5H), 7.53–7.70 (m, 2H), 8.61–8.79 (m, 1H), 8.99–9.20 (m, 1H). 

#### 4.4.82. 3-[2-(Benzyloxy)phenyl]-3-(2-methylpropyl)-4,5,6,7-tetrahydrofuro[3,4-c]pyridin-1(3H)-one (**87**)

The reduced analog was synthesized from pyridine **86** using procedure C to yield the desired compound (0.096 g, 0.52 mmol, 25% yield). ^1^H NMR (300 MHz, CDCl_3_) δ ppm 0.73–0.92 (m, 6H), 1.36–1.64 (m, 3H), 2.11–2.23 (m, 2H), 2.50–2.65 (m, 1H), 2.70–2.86 (m, 1H), 2.86–3.01 (m, 1H), 3.36–3.57 (m, 2H), 4.96–5.11 (m, 2H), 6.89–7.06 (m, 1H), 7.23–7.34 (m, 2H), 7.37–7.47 (m, 5H), 7.50–7.63 (m, 1H). ESI MS *m/z*: Calculated for C_24_H_27_NO_3_ 377.20, Found 378.3 (M + H)^+^.

#### 4.4.83. 3-(2-Hydroxyphenyl)-3-(2-methylpropyl)-4,5,6,7-tetrahydrofuro[3,4-c]pyridin-1(3H)-one (**88**)

Pd on carbon (10 mol%) was added to amine **87** (0.064 g, 0.17 mmol, 1 equiv.) in methanol (5 mL, 0.03 M). The reaction was put under hydrogen at 50 psi for 1 h. The reaction was filtered and carried forward crude. 

#### 4.4.84. 3-[1-(3-Chlorophenyl)-1-hydroxy-3-methylbutyl]-N,N-bis(1-methylethyl)pyridine-4-carboxamide (**89**)

The alcohol intermediate was synthesized from N,N-diisopropylisonicotinamide and 1-(3-chlorophenyl)-3-methyl-1-butanone according to procedure A to yield the desired compound (1.8 g, 4.02 mmol, 50% yield). ^1^H NMR (300 MHz, CDCl_3_) δ ppm 0.40–0.50 (m, 3H), 0.56–0.66 (m, 3H), 0.96–1.02 (m, 4H), 1.09 (d, *J* = 6.40 Hz, 3H), 1.20–1.29 (m, 3H), 1.36–1.43 (m, 3H), 1.50–1.70 (m, 2H), 1.97–2.06 (m, 1H), 2.22–2.38 (m, 1H), 3.09–3.35 (m, 2H), 6.34–6.45 (m, 1H), 6.99–7.09 (m, 1H), 7.13–7.40 (m, 3H), 8.53–8.61 (m, 1H), 8.99 (s, 1H). 

#### 4.4.85. 3-(3-Chlorophenyl)-3-(2-methylpropyl)furo[3,4-c]pyridin-1(3H)-one (**90**)

The pyridine intermediate was synthesized from alcohol **89** using procedure B to yield the desired compound (0.50 g, 1.66 mmol, 41% yield). ^1^H NMR (300 MHz, CDCl_3_) δ ppm 0.78–0.94 (m, 6H), 1.43–1.53 (m, 1H), 1.53–1.72 (m, 2H), 2.08–2.14 (m, 1H), 2.50 (dd, *J* = 15.07, 5.27 Hz, 1H), 7.22–7.40 (m, 2H), 7.42–7.49 (m, 1H), 7.53–7.59 (m, 1H), 7.74–7.84 (m, 1H), 8.87 (d, *J* = 4.90 Hz, 1H), 9.06 (d, *J* = 1.13 Hz, 1H). 

#### 4.4.86. 3-(3-Chlorophenyl)-3-(2-methylpropyl)-4,5,6,7-tetrahydrofuro[3,4-c]pyridin-1(3H)-one (**91**)

The reduced analog was synthesized from pyridine **90** using procedure C to yield the desired compound (310 mg, 1.01 mmol, 61% yield). ^1^H NMR (300 MHz, CDCl_3_) δ ppm 0.82–1.02 (m, 6H), 1.55–1.84 (m, 3H), 2.14–2.40 (m, 3H), 2.95 (tdd, *J* = 18.32, 12.90, 5.84 Hz, 2H), 3.37–3.52 (m, 1H), 3.62–3.73 (m, 1H), 7.15–7.23 (m, 1H), 7.26–7.39 (m, 3H). 

#### 4.4.87. Tert-Butyl 4-Benzylpiperazine-1-carboxylate (**92**)

Boc_2_O (2.43 g, 11.1 mmol, 1.2 equiv.) and triethyl amine (6.45 mL, 46.5 mmol, 5 equiv.) were added to 1-(phenylmethyl)-piperazine dihydrochloride (1.98 g, 9.3 mmol, 1 equiv.) in THF (40 mL, 0.2 M). The reaction was stirred until complete by TLC. The reaction was quenched with water, diluted with EtOAc, and extracted. The organic layer was dried with sodium sulfate, filtered, and concentrated. The resulting residue was purified by column chromatography to yield the desired Boc-protected piperazine (1.12 g, 4.0 mmol, 43% yield). ^1^H NMR (300 MHz, CDCl_3_) δ ppm 1.45 (s, 9H), 2.25–2.45 (m, 4H), 3.35–3.46 (m, 4H), 3.50 (s, 2H), 7.01–7.55 (m, 5H).

#### 4.4.88. 7-Benzyl-1-(2-methylpropyl)-1-phenylhexahydro[1,3]oxazolo[3,4-a]pyrazin-3-one (**93**)

TMEDA (1 mL, 6.6 mmol, 2.2 equiv.), sec-butyl lithium (4.7 mL, 1.4 M solution, 6.6 mmol, 2 equiv.) and isovalerophenone (0.97 g, 6.0 mmol, 2 equiv.) were added to piperazine **92** (830 mg, 3.0 mmol, 1 equiv.) in THF (10 mL, 0.3 M). The reaction was monitored by TLC, and upon completion, was quenched with aqueous sodium bicarbonate, diluted with EtOAc, and extracted. The organic layer was dried with sodium sulfate, filtered, and concentrated. The resulting residue was purified by column chromatography to yield the desired compound (324 mg, 0.89 mmol, 30% yield). ^1^H NMR (300 MHz, CDCl_3_) δ ppm 0.64 (d, *J* = 6.78 Hz, 3H), 0.91 (d, *J* = 6.59 Hz, 3H), 1.80 (d, *J* = 6.03 Hz, 2H), 2.04–2.15 (m, 1H), 2.24 (t, *J* = 10.83 Hz, 1H), 2.72 (dd, *J* = 11.49, 3.39 Hz, 1H), 2.83–3.09 (m, 2H), 3.60 (q, *J* = 13.25 Hz, 2H), 3.69–3.82 (m, 2H), 6.99–7.63 (m, 10H).

#### 4.4.89. 9H-Fluoren-9-ylmethyl 1-(2-methylpropyl)-3-oxo-1-phenyltetrahydro[1,3]oxaZolo[3,4-a]pyrazine-7(1H)-carboxylate (**94**)

Fmoc-Cl (0.17 g, 0.66 mmol, 1.1 equiv.) was added to oxazolidinone **93** (220 mg, 0.60 mmol, 1 equiv.) in acetonitrile (10 mL, 0.06 M). The reaction was heated to 90 °C for 4 h. The reaction was quenched with aqueous sodium bicarbonate, diluted with EtOAc, and extracted. The organic layer was dried with sodium sulfate, filtered, and concentrated. The residue was carried forward crude. 

#### 4.4.90. 1-(2-Methylpropyl)-1-phenylhexahydro[1,3]oxazolo[3,4-a]pyrazin-3-one (**95**)

Piperidine (0.1 mL, 0.90 mmol, 1.5 equiv.) was added to oxazolidinone **94** (30 mg, 0.6 mmol, 1 equiv.) in acetonitrile (5 mL, 0.01 M). The reaction was stirred until complete by TLC. The reaction was quenched with aqueous sodium bicarbonate, diluted with EtOAc, and extracted. The organic layer was separated, dried with sodium sulfate, filtered, and concentrated. The residue was purified by column chromatography to yield the desired amine (164 mg, 0.6 mmol, quantitative yield). ^1^H NMR (300 MHz, CDCl_3_) δ ppm 0.70 (d, *J* = 6.78 Hz, 3H), 0.86–0.99 (m, 3H), 1.51–1.70 (m, 1H), 1.83 (t, *J* = 11.59 Hz, 1H), 1.93–2.11 (m, 2H), 2.41–2.62 (m, 2H), 2.84 (dd, *J* = 11.96, 3.67 Hz, 1H), 3.06 (ddd, *J* = 13.00, 12.06, 3.77 Hz, 1H), 3.60 (dd, *J* = 11.11, 3.58 Hz, 1H), 3.83 (dd, *J* = 13.09, 2.92 Hz, 1H), 7.03–7.58 (m, 5H).

### 4.5. Ca^+2^ Mobilization Assay 

A stable cell line overexpressing the human NPSR-107I was created in CHO-Gα_q16_ cells (RD-HGA16, Molecular Devices), as previously described [10]. Briefly, CHO-Gα_q16_ cells were transfected with pcDNA 3.1 human NPSR-107I using Lipofectamine reagent. Transfected cells were plated into 10 cm^2^ dishes 24 h post-transfection and were selected using Geneticin. After 2 weeks, individual clones were selected, grown to confluence, and screened in a functional 96-well calcium mobilization assay that measured the response to NPS (100 nM final). Concentration–response curves of NPS were run in clones that showed a high response to the agonist in the screen. A final working clone was chosen for all subsequent studies based on NPS potency and the calcium mobilization signal window. CHO-Gα16-hNPSR-107I cells were cultured in Ham’s F12 media supplemented with 10% FBS, 100 units/mL penicillin, 100 μg/mL streptomycin, 200 μg/mL hygromycin, and 400 μg/mL geneticin. Media were purchased from HyClone (GE Life Sciences); FBS, penicillin/streptomycin, hygromycin, and geneticin were purchased from Invitrogen (Thermo Scientific). The day before the assay, cells were plated into 96-well black-walled assay plates at 25,000–40,000 cells/well in growth medium without selection antibiotics. Cells were incubated overnight at 37 °C, 5% CO_2_. The FLIPR Calcium 5 assay kit (Molecular Devices) was used according to the manufacturer’s instructions with minor modifications. Briefly, reconstituted dye was diluted in pre-warmed assay buffer (HBSS, 20 mM HEPES, 2.5 mM probenecid, pH 7.4 at 37 °C). Growth medium was removed, and the cells were gently washed with assay buffer. Calcium 5 dye was added to the cells and the plate was incubated for 45 min at 37 °C, 5% CO_2_. A single concentration of test compound and 10-point concentration–response curves of NPS (ranging from 100 nM to 1 pM) were prepared at 10× the desired final concentration. Cells were pretreated with the test compound or vehicle in a final concentration of 0.25% BSA/1% DMSO for 15 min at 37 °C. Assay plates were read with a FlexStation II instrument (excitation at 485 nm, detection at 525 nm, Molecular Devices). Calcium-mediated changes in fluorescence were monitored every 1.5 s over a 90 s time period. NPS was added during the read and the maximum kinetic reduction (SoftMax [Max-Min], Molecular Devices) in relative fluorescent units (RFU) was measured. Data were fitted to a three-parameter logistic curve to generate EC_50_ values (GraphPad Prism 6.0). Antagonism was quantified by measuring the ratio of equi-active concentrations of agonist measured in the presence or absence of the antagonist. Apparent Ke values were calculated using the equation Ke = [L]/((EC_50_^+^/EC_50_^−^) − 1), where [L] is the concentration of test compound, EC_50_^+^ is the EC_50_ of NPS with test compound, and EC_50_^–^ is the EC_50_ of NPS alone. Ke values were considered valid when the EC_50_^+^/EC_50_^−^ ratio was at least 4. HBSS and HEPES were purchased from Gibco (Thermo Scientific); probenecid, BSA, and DMSO were purchased from Sigma-Aldrich.

### 4.6. Animal Studies

Male mice between 8 and 12 weeks of age were used for all tests. C57/Bl6 were obtained from the former NCI colony maintained by Charles River. Animals were housed with ad libitum access to food and water on a 12:12 h light:dark cycle with lights on at 06:00. Experiments were conducted between the hours of 09:00 and 15:00. All experiments were approved by the Institutional Animal Care and Use Committee of the State University of New York at Buffalo (PMY09073N) and conducted in accordance with the National Institutes of Health Guide for the Care and Use of Laboratory animals.

### 4.7. Pharmacokinetics

Animals were administered vehicle (1% n-methyl-2-pyrrolidone (NMP) with 0.5% Tween 80 in 0.5% carboxymethyl cellulose (CMC) in water) or compound **14b** dissolved in the same vehicle (30 mg/kg, i.p.), and were euthanized at the appropriate timepoints by isoflurane overdose with decapitation. Trunk blood and brains were taken at 15, 30, 60, 120, and 240 min post-injection. Blood was collected in 5 mL tubes that had been preloaded with 0.5 M EDTA so that the final concentration would be approximately 5 mM. Blood/EDTA was centrifuged at 1000× *g* at 4 °C for 10 min. Plasma was collected and flash-frozen on dry ice and stored at −80 °C until analysis. 

### 4.8. Standard Curve Preparation

Stock solutions of **14b** were prepared and diluted serially to generate standard curve and quality control spiking solutions. The concentrations in plasma and brain homogenate for **14b** were 0.5, 1, 5, 10, 50, 100, 500, and 1000 ng/mL for standard curves. 

### 4.9. Sample Preparation

Brains were weighed and homogenized at 1:5 (wt:vol) with 50:50 ethanol:water using a Geno/Grinder 2010 (SPEXSamplePrep, Metuchen, NJ, USA) twice at 1750 rpm for 30 s. Subsequently, 50 µL of plasma or brain homogenate was mixed with 150 µL of acetonitrile containing reserpine as internal standard (100 ng/mL) in a 96-well plate. The plate was shaken at 700 rpm for 3 min and then centrifuged at 4000 rpm for 10 min at 4 C. Then, 50 µL of supernatant was mixed with 50 µL of water in a new plate and analyzed by LC/MS-MS.

### 4.10. LC/MS-MS Conditions

A SCIEX Exion AD UHPLC system (Framingham, MA) was used to inject 10 µL of the processed samples/calibration standards onto a Waters Acquity BEH C18 (2.1 mm × 100 mm, 1.7 µM) column (Milford, MA). Mobile phase A consisted of 0.1% formic acid in water, and mobile phase B consisted of 0.1% formic acid in acetonitrile. Chromatography was conducted using a linear gradient starting at initial conditions of 10% B, and holding for 0.5 min before increasing linearly to 95% B over 3.5 min, and holding for 1 min before returning to initial conditions over 0.1 min. Total run time was 7 min and the flow rate was 0.400 mL/min. Quantitation was achieved by multiple reaction monitoring in positive ion mode using an SCIEX 6500+ (Framingham, MA) QTRAP mass spectrometer. Compound **14b** was quantitated by monitoring the transition of *m*/*z* 445.044 → 251.1, and the internal standard, reserpine, was monitored by *m*/*z* 609.300 → 194.9. Mass spectrometry parameters were as follows: CUR = 30, GS1 = 50, GS2 = 50, TEM = 700, CAD = low, and IS = 4500. Calibration curves were processed using Analyst 1.7.1 software by plotting the analyte to internal standard peak area ratio against the calibration standard concentrations.

### 4.11. Cannula Implantation

Mice were anesthetized using ketamine (100 mg/kg) and xylazine (5 mg/kg) and were secured to a stereotaxic frame once the appropriate plane of anesthesia was reached. An incision was made in order to expose the skull. A hole was drilled and the cannulae were placed at AP of −0.4 mm from bregma ML of –1.0 mm from the midline and at a depth (DV) of −2.0 mm from the dura. The cannulae were then secured with Metabond dental cement (Parkell Inc., Edgewood, NY, USA). The incision was then closed around the cement with surgical staples. Dummy cannulae were placed in the cannula to prevent the entry of foreign material. Post-operative care included saline (1 mL, sc), enrofloxacin (5 mg/kg, sc), and carprofen (5 mg/kg, sc). All mice were placed under a heating lamp and observed until they were awake. For three additional days after surgery, mice were given carprofen (5 mg/kg, sc). They were housed in their home cages for one week to recover from surgery. 

### 4.12. Locomotor Activity Analysis

Open-field testing was conducted in clear plexiglass cages measuring 40 × 40 cm^2^ interfaced by a grid array of infrared beams connected to a computer system which tracked and quantified the animals’ location and movements (OMNITECH Instruments, Columbus, OH, USA). Testing was conducted in a room novel to the mice.

Mice were moved to the testing room and immediately placed in the testing boxes, where they remained undisturbed for 90 min. Each mouse was removed and administered vehicle or compound **14b** (3 mg/kg, i.p.), placed back into the test arena for 10 min before administering either artificial cerebral spinal fluid with 0.1% bovine serum albumin (aCSF) or NPS (0.3 nmole; Anaspec, Fremont, CA, USA) via the indwelling cannula in a volume of 2 microliters. Recording of locomotor activity began immediately after the ICV injection.

Statistical analyses were performed by ANOVA with Tukey’s multiple comparisons test analysis within GraphPad Prism 8 software (GraphPad Software, San Diego, CA, USA). All results are expressed as mean values ± SEM. Group means were considered significantly different when *p* < 0.05. 

### 4.13. Glutathione Incubation Assay

For the glutathione incubation assay, 20 mM stock solutions of compound **14b** in MeCN (400 µL) and glutathione GSH in MeCN: Milli-Q H_2_O (10 mL, 1: 1, *v*/*v*) were prepared in separate scintillation vials. To prepare the incubation solution, 100 µL of **14b** solution and 900 µL of GSH solution were taken in a clean HPLC vial. The final concentrations of **14b** and GSH were 2 mM and 18 mM, respectively. In addition, a 2 mM reference solution of **14b** was prepared by adding 100 µL of **14b** stock solution to an HPLC vial containing 900 µL of MeCN: H_2_O (1:1), and an 18 mM reference solution of GSH was prepared by adding 900 µL of GSH stock solution to an HPLC vial containing 100 µL of MeCN. 

Low-resolution mass spectra were obtained using an Agilent InfinityLab MSD single quadrupole mass spectrometer system (ESI). Mass detection was carried out both in positive and negative mode ESI at 200–2000 *m*/*z* range. The reference solutions of **14b** and GSH were injected once, individually. Consecutive injections were submitted from the incubation solution (**14** + GSH) at t = 5 min, 1 h, 2 h, 6 h, 24 h, and 48 h. Chromatograms were detected using 254 and 280 nm wavelengths with 5 µL injections and data were processed using a general walkup method*. 

*LC/MS Walkup Method: an Agilent InfinityLab MSD single quadrupole mass spectrometer equipped with an API-ES and an Agilent Infinity II 1260 HPLC equipped with an Agilent Infinity 1260 variable wavelength detector and a Phenomenex Synergi 2.5 mm Hydro-RP 100A C18 30 × 2 mm column. HPLC Method: starting with a flow rate of 0.6 mL/min for 0.4 min at 20% solvent B followed by a 1.6-min gradient of 20–95% solvent B at 0.6 mL/min followed by 2 min at 95% solvent B with a gradual ramp-up of the flow rate to 1.2 mL/min at the end (solvent A, water with 0.1% formic acid; solvent B, acetonitrile with 0.1% formic acid and 5% water; absorbance monitored at 220 and 280 nm). MS Method: using atmospheric pressure ionization–electrospray, positive and negative ions were monitored in the range of 70–700 or 200–2000 (for compounds with a MW > 700).

## 5. Conclusions

This study was designed to evaluate a new series of NPSR antagonists and provide a deeper understanding of the key features of the SHA scaffold that lead to high potency at the NPSR. The diaryl system of SHA appears to be required for high antagonist potency when the oxazolidinone core is intact. Once the oxazolidinone nitrogen is replaced with a double bond, the binding mode appears to shift in the NPSR binding site and aliphatic groups are now tolerated for one of the aryl rings. Comparison of compound **29** with **14b** demonstrates that the binding sites for these two series are distinct. It was anticipated that the replacement of one aryl ring would enhance the solubility and drug-like properties of this class. Compound **14b** was selected for further study based on antagonist potency and the potential to exhibit differing physiochemical properties in vivo. Pharmacokinetic evaluation of **14b** indicated that it accesses the CNS in sufficient quantities but is cleared rapidly from plasma, suggesting the need to reduce oxidative metabolism. Behavioral evaluation of **14b** in a mouse model of NPS-stimulated locomotor activity demonstrated that a relatively low dose of **14b** (3 mg/kg i.p.) was sufficient to significantly decrease NPS (0.3 nmol)-mediated locomotor stimulation. Although **14b** is approximately sevenfold less potent than SHA-68, it possessed enhanced in vivo activity compared to published reports. Typically, SHA-68 is dosed at 50 mg/kg to significantly block 0.1 nmol of NPS-stimulated locomotor activity [12,20]. In this study, a significant reduction in NPS-mediated locomotor activity with a reduced dose of **14b** (3 mg/kg) and a threefold higher dose of NPS (0.3 nmol) was achieved. Although it is difficult to compare behavioral results across different labs, our data suggest that further improvements in potency and clearance of **14b** should result in substantially improved antagonists for the NPSR. Overall, we have now identified a novel NPSR antagonist scaffold with enhanced properties in vivo. 

## Figures and Tables

**Table 1:** Modifications of the core oxazolidinone ring.

Compound No.	Structure	K_e_ (nM) ^a^	cLogP	TPSA [Å^2^]
**1**	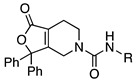 R = Bn	522	4.27	58.64
**2**	R = *n*-Bu	515	3.86	58.64
**3**	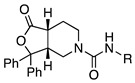 R = Bn	761	4.03	58.64
**4**	R = *n*-Bu	NA	3.63	58.64
**5**	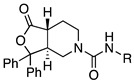 R = Bn	170	4.03	58.64
**6**	R = *n*-Bu	260	3.63	58.64
**7**	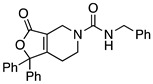	1133	4.27	58.64

^a^ Calcium Mobilization.

**Table 2:** Modifications of the diaryl motif.

Compound No.	Structure	K_e_ (nM) ^a^	cLogP	TPSA [Å^2^]
**8**	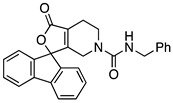	NA	3.94	58.64
**9**	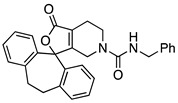	1655	4.83	58.64
**10**	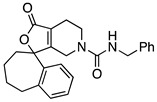	NA	4.05	58.64
**11**	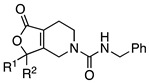 R^1^ = *n*-Bu, R^2^ = Ph	165	4.45	58.64
**12**	R^1^ = Bn, R^2^ = Ph	606	4.69	58.64
**13**	R^1^ = *neo*-pentyl, R^2^ = Ph	146	4.59	58.64
**14**	R^1^ = *iso*-Bu, R^2^ = Ph	118	4.29	58.64
**15**	R^1^ = Me, R^2^ = Ph	NA	3.04	58.64
**16**	R^1^, R^2^ = *iso*-Bu	734	4.18	58.64
**17**	R^1^ = Me_2_NCH_2_CH_2_, R^2^ = Ph	NA	2.76	61.88
**18**	R^1^ = CH_2_OH, R^2^ = Ph	NA	1.99	78.87
**19**	R^1^ = CH_2_OAc, R^2^ = Ph	NA	2.43	84.94
**20**	R^1^ = Me, R^2^ = 3-OH-Ph	NA	2.73	78.87
**21**	R^1^ = Me, R^2^ = 3-OBn-Ph	NA	4.60	67.87
**22**	R^1^ = *iso*-Bu, R^2^ = 2-Naphthyl	500	5.28	58.64
**23**	R^1^ = *iso*-Bu, R^2^ = 2-OH-Ph	1500	3.99	78.87
**24**	R^1^ = *iso*-Bu, R^2^ = 2-OBn-Ph	572	5.86	67.87
**25**	R^1^ = *iso*-Bu, R^2^ = 3-Cl-Ph	136	4.89	58.64

^a^ Calcium Mobilization.

**Table 3:** Modifications of urea portion of lead compound **14**.

Compound No.	Structure	K_e_ (nM) ^a^	cLogP	TPSA [Å^2^]
**14**	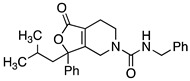	118	4.29	58.64
**14a**	(-) Isomer	432		
**14b**	(+) Isomer	71.4		
**26**	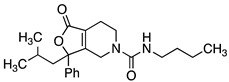	238	3.89	58.64
**27**	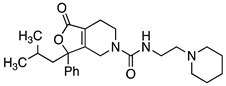	566	3.43	61.88
**28**	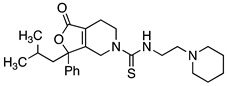	386	4.32	44.81
**29**	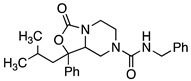	NA	3.93	61.88

^a^ Calcium Mobilization.

## Data Availability

Data available within article and Supplementary Materials.

## Supplementary Materials

The following are available online at https://www.mdpi.com/article/10.3390/ph14101024/s1, Figure S1: Compound **3** ^13^C-NMR, Figure S2. Compound **3** ^1^H-NMR, Figure S3. Compound **2**, Figure S4. Compound **3**, Figure S5. Compound **4**, Figure S6. Compound **5**, Figure S7. Compound **6**, Figure S8. Compound **7**, Figure S9. Compound **8**, Figure S10. Compound **9**, Figure S11. Compound **10**, Figure S12. Compound **11**, Figure S13. Compound **12**, Figure S14. Compound **13**, Figure S15. Compound **15**, Figure S16. Compound **16**, Figure S17. Compound **17**, Figure S18. Compound **18**, Figure S19. Compound **19**, Figure S20. Compound **20**, Figure S21. Compound **21**, Figure S22. Compound **22**, Figure S23. Compound **23**, Figure S24. Compound **24**, Figure S25. Compound **25**, Figure S26. Compound **26**, Figure S27. Compound **27**, Figure S28. Compound **28**, Figure S29. Compound **29**.
