# Supplementary material for: Identification of a Novel Neuropeptide S Receptor Antagonist Scaffold Based on the SHA-68 Core
Source: Pharmaceuticals (Basel). 2021 Oct 6;14(10):1024. doi: 10.3390/ph14101024 (PMC8538004; doi:10.3390/ph14101024)
Supplement: Supplementary file 1 [file pharmaceuticals-14-01024-s001.zip › pharmaceuticals-1346916 supplementary.pdf]

pharmaceuticals

MDPI

Supplementary Materials

Identification of a Novel Neuropeptide S Receptor Antagonist Scaffold based on the SHA-68 Core

Allison Zarkin [1], Rajwana Jahan [1], Rajendra Upretty [1], Yanan Zhang [1], Charles McElhinny [1], Rodney Snyder [1], Elaine Gay [1], Gabriel Jewula [2], Heather Bool [2], Stewart D Clark [2] and Scott Runyon [1,*]

[1] Research Triangle Institute, Post Office Box 12194, Research Triangle Park, NC 27709-2194, USA
[2] Department of Pharmacology and Toxicology, State University of New York at Buffalo, 3435 Main Street, Buffalo, NY, 14214, USA
* Correspondance: srunyon@rti.org

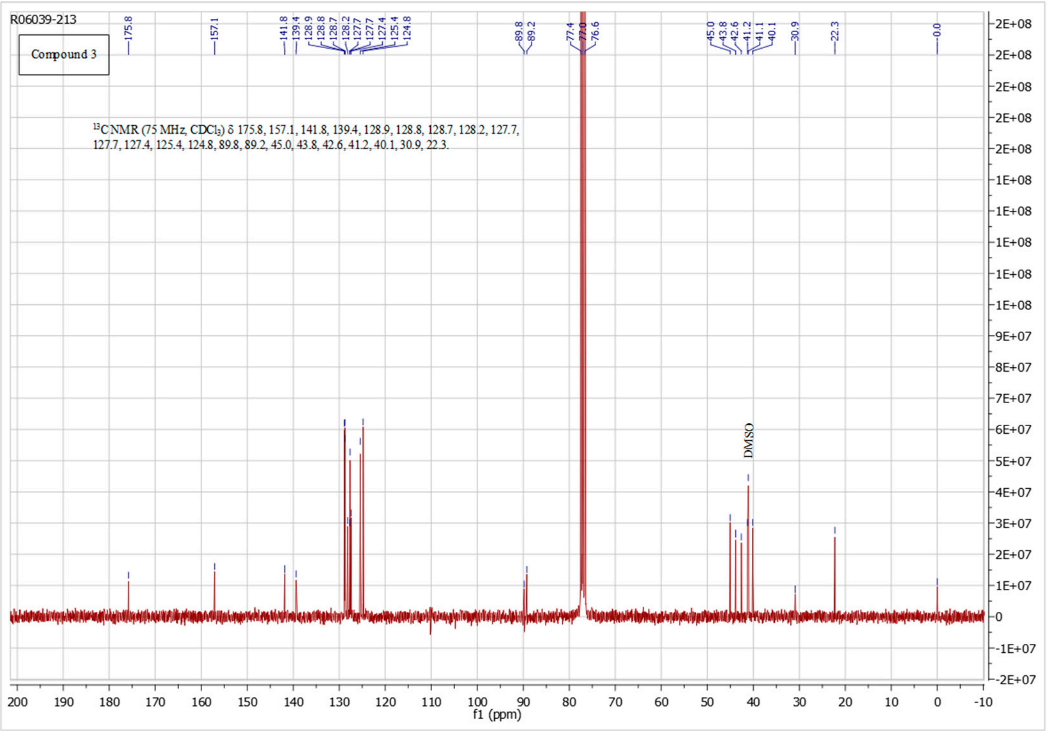

Figure S1. Compound 3 ^{13}C-NMR.

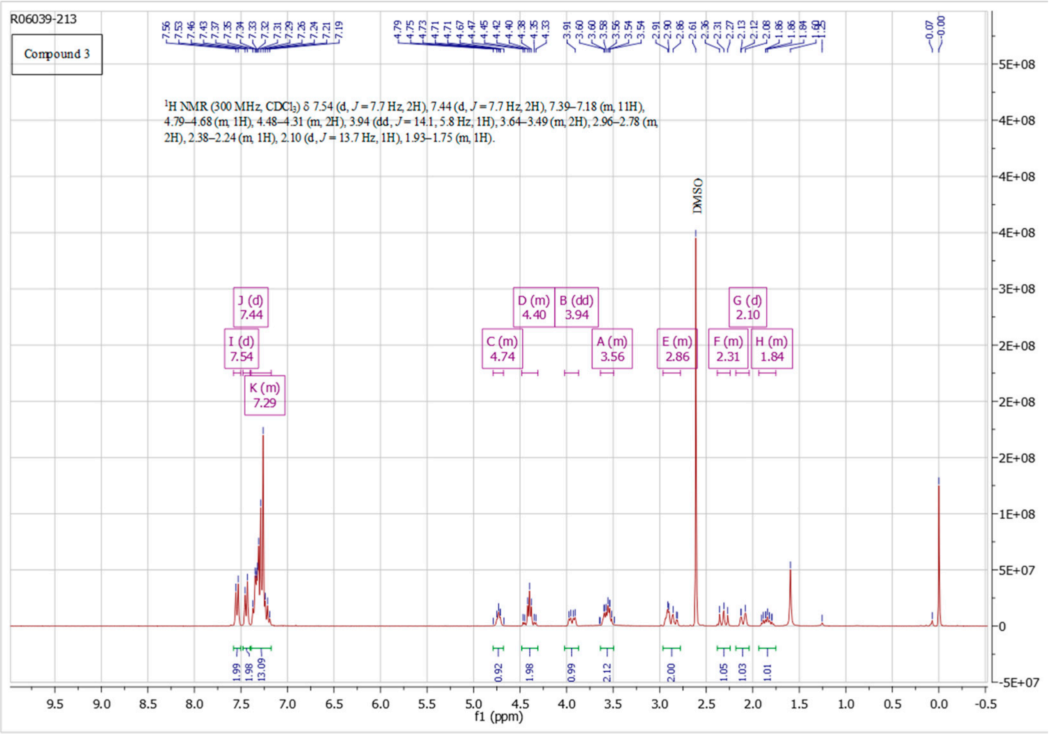

Figure S2. Compound 3 ^{1}H-NMR.

Empower 2 software — WalkUpReport

SAMPLE INFORMATION

Sample Name:	R06039-199	Acquired By:	walkup
Sample Type:	Unknown	Sample Set Name:	RajwanaR06039210423
Vial:	107	Acq. Method Set:	MethodSetAB5T95B15Min
Injection #:	1	Processing Method:	220W280W15MIN
Injection Volume:	10.00 ul	Channel Name:	W2996 220.0nm-2.0
Run Time:	30.0 Minutes	Proc. Chnl. Descr.:	W2996 PDA 220.0 nm at 2.0
Date Acquired:	4/23/2021 8:08:13 PM EST		
Date Processed:	4/23/2021 8:38:25 PM EST		

Auto-Scaled Chromatogram

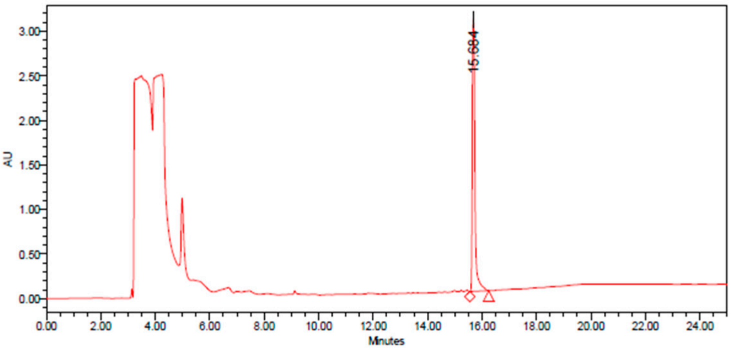

Peak Results

	RT	Area	% Area	Height
1	15.684	20389969	100.00	3111467

Reported by User: walkup
Report Method: WalkUpReport
Report Method ID: 1018
Page: 1 of 1

Project Name: MedChem\MedChem2021-04
Date Printed:
4/28/2021
2:31:13 PM America/New_York

Figure S3. Compound 2.

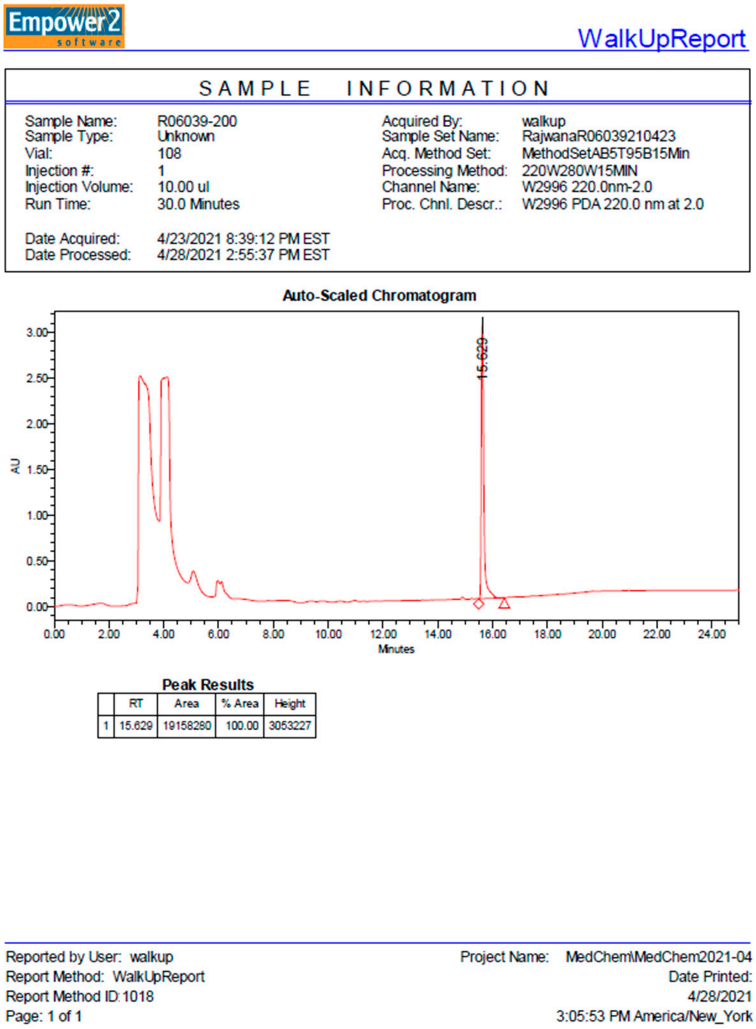
Empower 2 software

WalkUpReport

SAMPLE INFORMATION

Sample Name:	R06039-200	Acquired By:	walkup
Sample Type:	Unknown	Sample Set Name:	RajwanaR06039210423
Vial:	108	Acq. Method Set:	MethodSetAB5T95B15Min
Injection #:	1	Processing Method:	220W280W15MIN
Injection Volume:	10.00 ul	Channel Name:	W2996 220.0nm-2.0
Run Time:	30.0 Minutes	Proc. Chnl. Descr.:	W2996 PDA 220.0 nm at 2.0
Date Acquired:	4/23/2021 8:39:12 PM EST		
Date Processed:	4/28/2021 2:55:37 PM EST		

Auto-Scaled Chromatogram

Peak Results

	RT	Area	% Area	Height
1	15.629	19158280	100.00	3053227

Reported by User: walkup
Report Method: WalkUpReport
Report Method ID 1018
Page: 1 of 1

Project Name: MedChem\MedChem2021-04
Date Printed:
4/28/2021
3:05:53 PM America/New_York

Figure S4. Compound 3.

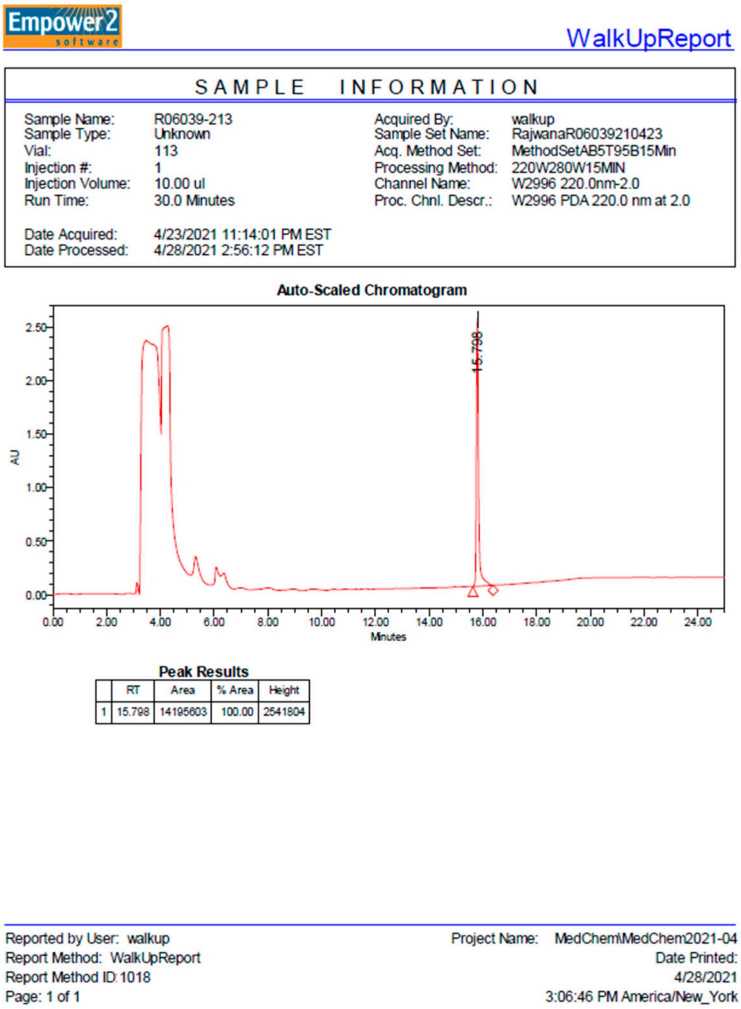

	RT	Area	% Area	Height
1	15.798	14195603	100.00	2541804

Figure S5. Compound 4.

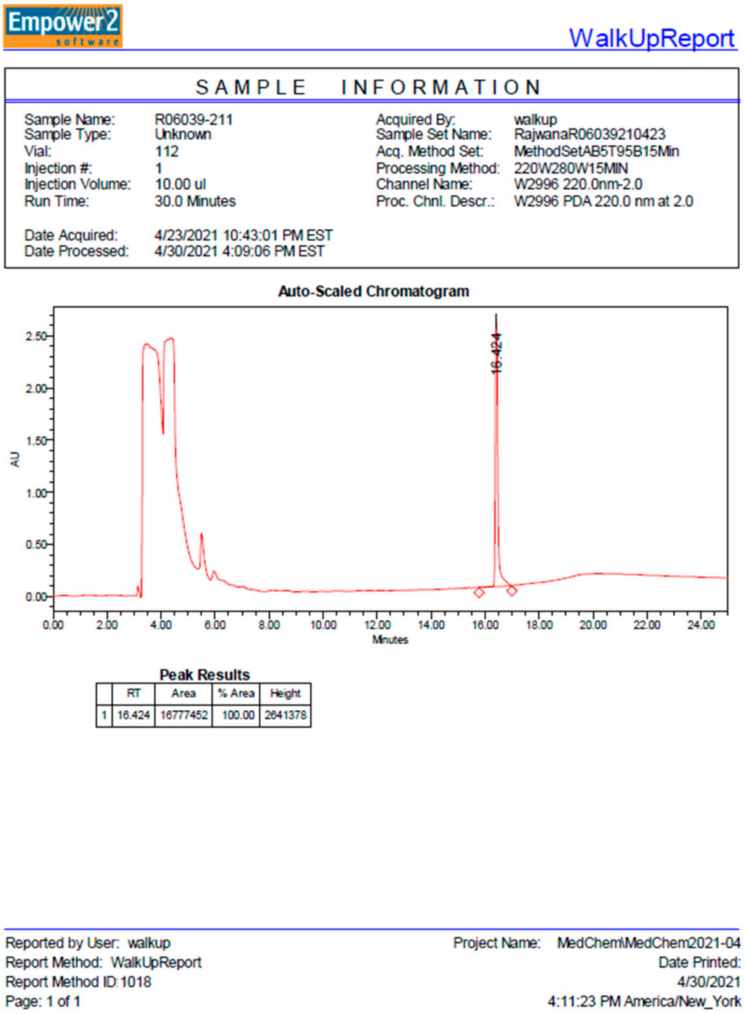
Empower2 software

WalkUpReport

SAMPLE INFORMATION

Sample Name:	R06039-211	Acquired By:	walkup
Sample Type:	Unknown	Sample Set Name:	RajwanaR06039210423
Vial:	112	Acq. Method Set:	MethodSetAB5T95B15Min
Injection #:	1	Processing Method:	220W280W15MIN
Injection Volume:	10.00 ul	Channel Name:	W2996 220.0nm-2.0
Run Time:	30.0 Minutes	Proc. Chnl. Descr.:	W2996 PDA 220.0 nm at 2.0
Date Acquired:	4/23/2021 10:43:01 PM EST		
Date Processed:	4/30/2021 4:09:06 PM EST		

Auto-Scaled Chromatogram

Peak Results

	RT	Area	% Area	Height
1	16.424	16777452	100.00	2641378

Reported by User: walkup
Report Method: WalkUpReport
Report Method ID: 1018
Page: 1 of 1

Project Name: MedChem\MedChem2021-04
Date Printed:
4/30/2021
4:11:23 PM America/New_York

Figure S6. Compound 5.

Empower2 software

WalkUpReport

SAMPLE INFORMATION

Sample Name:	R06039-214	Acquired By:	walkup
Sample Type:	Unknown	Sample Set Name:	RajwanaR06039214210505
Vial:	1	Acq. Method Set:	MethodSetAB5T95B15Min
Injection #:	1	Processing Method:	220W280W15MIN
Injection Volume:	20.00 ul	Channel Name:	W2996 220.0nm-2.0
Run Time:	30.0 Minutes	Proc. Chnl. Descr.:	W2996 PDA 220.0 nm at 2.0
Date Acquired:	5/5/2021 12:48:10 PM EST		
Date Processed:	5/5/2021 3:36:31 PM EST		

Auto-Scaled Chromatogram

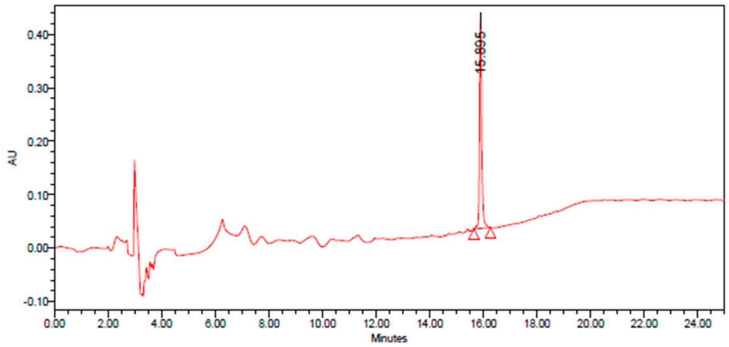

Peak Results

	RT	Area	% Area	Height
1	15.895	2183496	100.00	388062

Reported by User: walkup
Report Method: WalkUpReport
Report Method ID: 1018
Page: 1 of 1

Project Name: MedChem\MedChem2021-05
Date Printed:
5/5/2021
3:36:47 PM America/New_York

Figure S7. Compound 6.

WalkUpReport

SAMPLE INFORMATION

Sample Name:	R06039-212	Acquired By:	walkup
Sample Type:	Unknown	Sample Set Name:	RajwanaR06039212210429
Vial:	46	Acq. Method Set:	
Injection #:	1	Processing Method:	220W280W15MIN
Injection Volume:	20.00 ul	Channel Name:	W2996 220.0nm-2.0
Run Time:	30.0 Minutes	Proc. Chnl. Descr.:	W2996 PDA 220.0 nm at 2.0
Date Acquired:	4/29/2021 12:01:26 PM EST		
Date Processed:	4/29/2021 2:20:42 PM EST		

Auto-Scaled Chromatogram

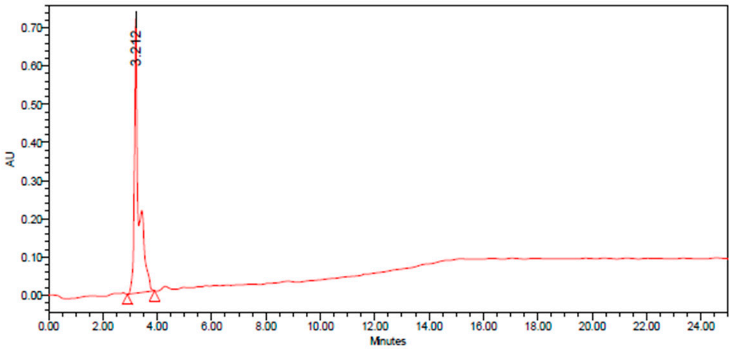

Peak Results

	RT	Area	% Area	Height
1	3.212	8080850	100.00	704970

Reported by User: walkup
Report Method: WalkUpReport
Report Method ID 1018
Page: 1 of 1

Project Name: MedChem\MedChem2021-04
Date Printed:
4/30/2021
3:59:59 PM America/New_York

Figure S8. Compound 7.

Empower2 software WalkUpReport

SAMPLE INFORMATION

Sample Name:	R06039-202	Acquired By:	walkup
Sample Type:	Unknown	Sample Set Name:	RajwanaR06039210423
Vial:	109	Acq. Method Set:	MethodSetAB5T95B15Min
Injection #:	1	Processing Method:	220W280W15MIN
Injection Volume:	10.00 ul	Channel Name:	W2996 220.0nm-2.0
Run Time:	30.0 Minutes	Proc. Chnl. Descr.:	W2996 PDA 220.0 nm at 2.0
Date Acquired:	4/23/2021 9:10:08 PM EST		
Date Processed:	4/28/2021 2:55:54 PM EST		

Auto-Scaled Chromatogram

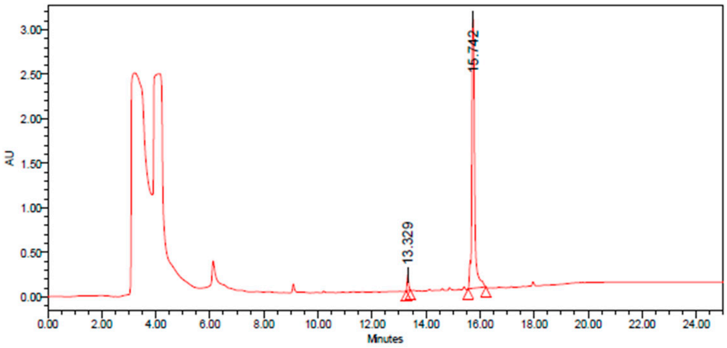

Peak Results

	RT	Area	% Area	Height
1	13.329	676802	2.91	170590
2	15.742	22542429	97.09	3145532

Reported by User: walkup
Report Method: WalkUpReport
Report Method ID: 1018
Page: 1 of 1

Project Name: MedChem\MedChem2021-04
Date Printed:
4/28/2021
3:06:18 PM America/New_York

Figure S9. Compound 8.

WalkUpReport

SAMPLE INFORMATION

Sample Name:	R06039-220	Acquired By:	walkup
Sample Type:	Unknown	Sample Set Name:	RajwanaR06039210423
Vial:	114	Acq. Method Set:	MethodSetAB5T95B15Min
Injection #:	1	Processing Method:	220W280W15MIN
Injection Volume:	10.00 ul	Channel Name:	W2996 220.0nm-2.0
Run Time:	30.0 Minutes	Proc. Chnl. Descr.:	W2996 PDA 220.0 nm at 2.0
Date Acquired:	4/23/2021 11:44:59 PM EST		
Date Processed:	4/28/2021 2:56:31 PM EST		

Auto-Scaled Chromatogram

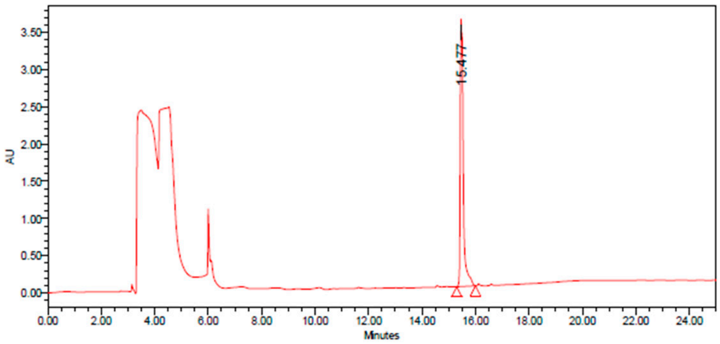

Peak Results

	RT	Area	% Area	Height
1	15.477	28411387	100.00	3566894

Reported by User: walkup
Report Method: WalkUpReport
Report Method ID 1018
Page: 1 of 1

Project Name: MedChem\MedChem2021-04
Date Printed:
4/28/2021
3:07:15 PM America/New_York

Figure S10. Compound 9.

Empower 2 software

WalkUpReport

SAMPLE INFORMATION

Sample Name:	R06039-221	Acquired By:	walkup
Sample Type:	Unknown	Sample Set Name:	RajwanaR06039210423
Vial:	115	Acq. Method Set:	MethodSetAB5T95B15Min
Injection #:	1	Processing Method:	220W280W15MIN
Injection Volume:	10.00 ul	Channel Name:	W2996 220.0nm-2.0
Run Time:	30.0 Minutes	Proc. Chnl. Descr.:	W2996 PDA 220.0 nm at 2.0
Date Acquired:	4/24/2021 12:15:59 AM EST		
Date Processed:	4/30/2021 4:09:25 PM EST		

Auto-Scaled Chromatogram

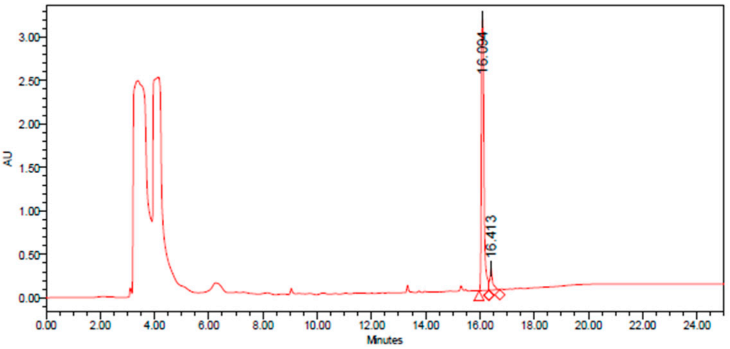

Peak Results

	RT	Area	% Area	Height
1	16.094	20506549	91.99	3194295
2	16.413	1786777	8.01	245718

Reported by User: walkup
Report Method: WalkUpReport
Report Method ID: 1018
Page: 1 of 1

Project Name: MedChem\MedChem2021-04
Date Printed:
4/30/2021
4:11:52 PM America/New_York

Figure S11. Compound 10.

Empower2 software WalkUpReport

SAMPLE INFORMATION

Sample Name:	R06039-233	Acquired By:	walkup
Sample Type:	Unknown	Sample Set Name:	RajwanaR06039210423
Vial:	11	Acq. Method Set:	MethodSetAB5T95B15Min
Injection #:	1	Processing Method:	220W280W15MIN
Injection Volume:	10.00 ul	Channel Name:	W2996 220.0nm-2.0
Run Time:	30.0 Minutes	Proc. Chnl. Descr.:	W2996 PDA 220.0 nm at 2.0
Date Acquired:	4/24/2021 3:22:05 AM EST		
Date Processed:	4/28/2021 2:48:39 PM EST		

Auto-Scaled Chromatogram

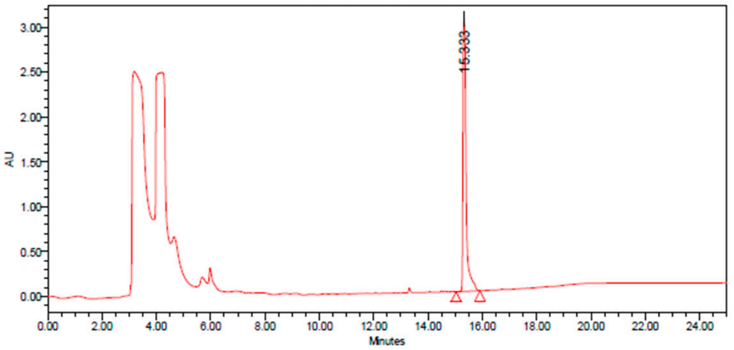

Peak Results

	RT	Area	% Area	Height
1	15.333	24465020	100.00	3042853

Reported by User: walkup
Report Method: WalkUpReport
Report Method ID 1018
Page: 1 of 1

Project Name: MedChem\MedChem2021-04
Date Printed:
4/28/2021
3:01:09 PM America/New_York

Figure S12. Compound 11.

Empower 2 software

WalkUpReport

SAMPLE INFORMATION

Sample Name:	R06039-224	Acquired By:	walkup
Sample Type:	Unknown	Sample Set Name:	RajwanaR06039210423
Vial:	116	Acq. Method Set:	MethodSetAB5T95B15Min
Injection #:	1	Processing Method:	220W280W15MIN
Injection Volume:	10.00 ul	Channel Name:	W2996 220.0nm-2.0
Run Time:	30.0 Minutes	Proc. Chnl. Descr.:	W2996 PDA 220.0 nm at 2.0
Date Acquired:	4/24/2021 12:46:53 AM EST		
Date Processed:	4/24/2021 1:17:06 AM EST		

Auto-Scaled Chromatogram

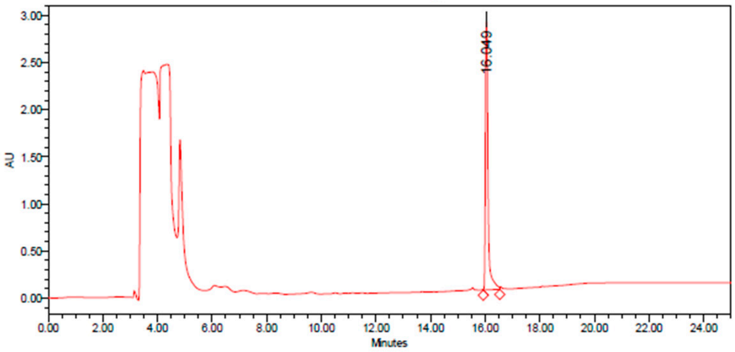

Peak Results

	RT	Area	% Area	Height
1	16.049	19125876	100.00	2934973

Reported by User: walkup
Report Method: WalkUpReport
Report Method ID 1018
Page: 1 of 1

Project Name: MedChem\MedChem2021-04
Date Printed:
4/28/2021
2:41:04 PM America/New_York

Figure S13. Compound 12

Empower2 software — WalkUpReport

SAMPLE INFORMATION

Sample Name:	R06039-225	Acquired By:	walkup
Sample Type:	Unknown	Sample Set Name:	RajwanaR06039210423
Vial:	117	Acq. Method Set:	MethodSetAB5T95B15Min
Injection #:	1	Processing Method:	220W280W15MIN
Injection Volume:	10.00 ul	Channel Name:	W2996 220.0nm-2.0
Run Time:	30.0 Minutes	Proc. Chnl. Descr.:	W2996 PDA 220.0 nm at 2.0
Date Acquired:	4/24/2021 1:17:51 AM EST		
Date Processed:	4/24/2021 1:48:03 AM EST		

Auto-Scaled Chromatogram

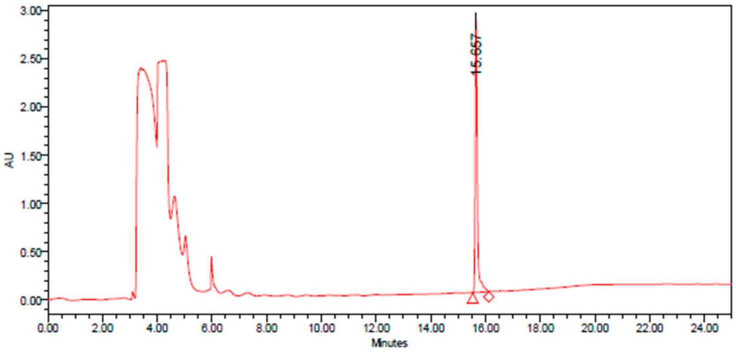

Peak Results

	RT	Area	% Area	Height
1	15.657	15401149	100.00	2888391

Reported by User: walkup
Report Method: WalkUpReport
Report Method ID: 1018
Page: 1 of 1

Project Name: MedChem\MedChem2021-04
Date Printed:
4/28/2021
2:43:46 PM America/New_York

Figure S14. Compound 13.

Empower2 software WalkUpReport

SAMPLE INFORMATION

Sample Name:	R06039-245	Acquired By:	walkup
Sample Type:	Unknown	Sample Set Name:	RajwanaR06039210423
Vial:	100	Acq. Method Set:	MethodSetAB5T95B15Min
Injection #:	1	Processing Method:	220W280W15MIN
Injection Volume:	10.00 ul	Channel Name:	W2996 220.0nm-2.0
Run Time:	30.0 Minutes	Proc. Chnl. Descr.:	W2996 PDA 220.0 nm at 2.0
Date Acquired:	4/23/2021 4:31:33 PM EST		
Date Processed:	4/28/2021 2:55:06 PM EST		

Auto-Scaled Chromatogram

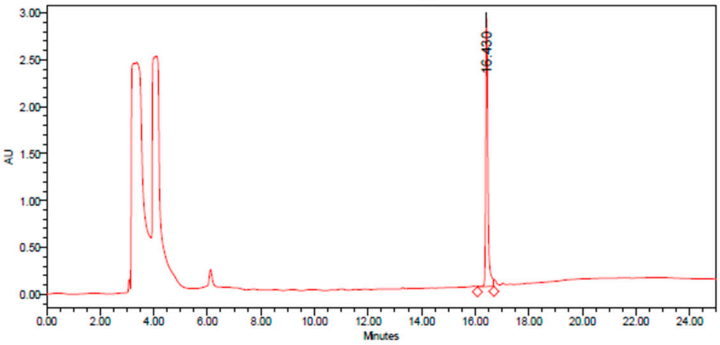

Peak Results

	RT	Area	% Area	Height
1	16.430	15931816	100.00	2882149

Reported by User: walkup
Report Method: WalkUpReport
Report Method ID: 1018
Page: 1 of 1

Project Name: MedChem\MedChem2021-04
Date Printed:
4/28/2021
3:04:56 PM America/New_York

Figure S15. Compound 15.

Empower2 software

WalkUpReport

SAMPLE INFORMATION

Sample Name:	R06039-230	Acquired By:	walkup
Sample Type:	Unknown	Sample Set Name:	RajwanaR06039210423
Vial:	119	Acq. Method Set:	MethodSetAB5T95B15Min
Injection #:	1	Processing Method:	220W280W15MIN
Injection Volume:	10.00 ul	Channel Name:	W2996 220.0nm-2.0
Run Time:	30.0 Minutes	Proc. Chnl. Descr.:	W2996 PDA 220.0 nm at 2.0
Date Acquired:	4/24/2021 2:19:53 AM EST		
Date Processed:	4/28/2021 2:45:38 PM EST		

Auto-Scaled Chromatogram

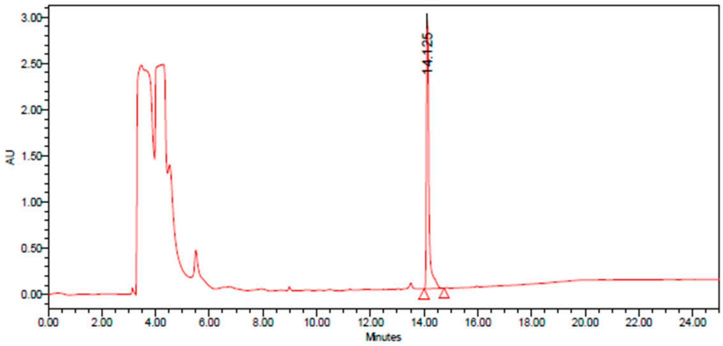

Peak Results

	RT	Area	% Area	Height
1	14.125	19283477	100.00	3059085

Reported by User: walkup
Report Method: WalkUpReport
Report Method ID: 1018
Page: 1 of 1

Project Name: MedChem\MedChem2021-04
Date Printed:
4/28/2021
2:57:24 PM America/New_York

Figure S16. Compound 16.

Empower2 software WalkUpReport

SAMPLE INFORMATION

Sample Name:	R06039-270	Acquired By:	walkup
Sample Type:	Unknown	Sample Set Name:	RajwanaR06039210423
Vial:	106	Acq. Method Set:	MethodSetAB5T95B15Min
Injection #:	1	Processing Method:	220W280W15MIN
Injection Volume:	10.00 ul	Channel Name:	W2996 220.0nm-2.0
Run Time:	30.0 Minutes	Proc. Chnl. Descr.:	W2996 PDA 220.0 nm at 2.0
Date Acquired:	4/23/2021 7:37:19 PM EST		
Date Processed:	4/30/2021 4:11:05 PM EST		

Auto-Scaled Chromatogram

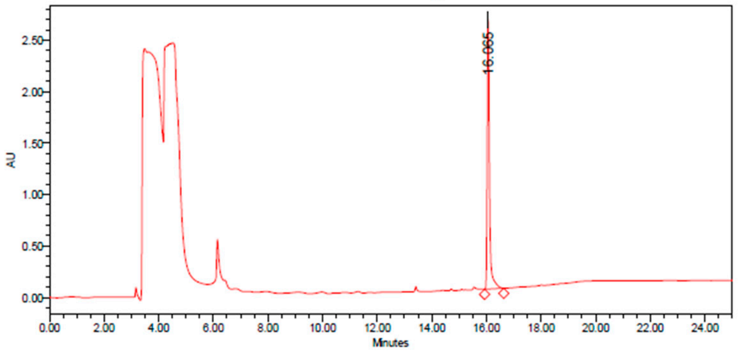

Peak Results

	RT	Area	% Area	Height
1	16.065	15509369	100.00	2661710

Reported by User: walkup
Report Method: WalkUpReport
Report Method ID: 1018
Page: 1 of 1

Project Name: MedChem\MedChem2021-04
Date Printed:
4/30/2021
4:12:49 PM America/New_York

Figure S17. Compound 17.

Empower2 software

WalkUpReport

SAMPLE INFORMATION

Sample Name:	R06039-231	Acquired By:	walkup
Sample Type:	Unknown	Sample Set Name:	RajwanaR06039210423
Vial:	120	Acq. Method Set:	MethodSetAB5T95B15Min
Injection #:	1	Processing Method:	220W280W15MIN
Injection Volume:	10.00 ul	Channel Name:	W2996 220.0nm-2.0
Run Time:	30.0 Minutes	Proc. Chnl. Descr.:	W2996 PDA 220.0 nm at 2.0
Date Acquired:	4/24/2021 2:50:49 AM EST		
Date Processed:	4/28/2021 2:47:59 PM EST		

Auto-Scaled Chromatogram

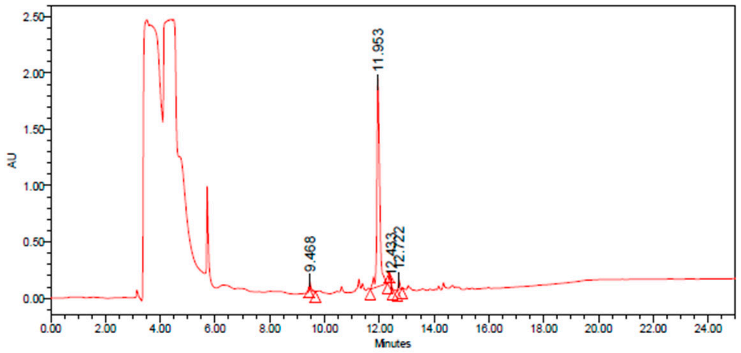

Peak Results

	RT	Area	% Area	Height
1	9.468	231154	1.69	56390
2	11.953	12998008	94.85	1868951
3	12.433	125353	0.91	-33885
4	12.722	349655	2.55	83931

Reported by User: walkup
Report Method: WalkUpReport
Report Method ID: 1018
Page: 1 of 1

Project Name: MedChem\MedChem2021-04
Date Printed:
4/28/2021
3:00:30 PM America/New_York

Figure S18. Compound 18.

Empower 2 software

WalkUpReport

SAMPLE INFORMATION

Sample Name:	R06039-240	Acquired By:	walkup
Sample Type:	Unknown	Sample Set Name:	RajwanaR06039210423
Vial:	14	Acq. Method Set:	MethodSetAB5T95B15Min
Injection #:	1	Processing Method:	220W280W15MIN
Injection Volume:	10.00 ul	Channel Name:	W2996 220.0nm-2.0
Run Time:	30.0 Minutes	Proc. Chnl. Descr.:	W2996 PDA 220.0 nm at 2.0
Date Acquired:	4/24/2021 4:54:59 AM EST		
Date Processed:	4/28/2021 3:02:59 PM EST		

Auto-Scaled Chromatogram

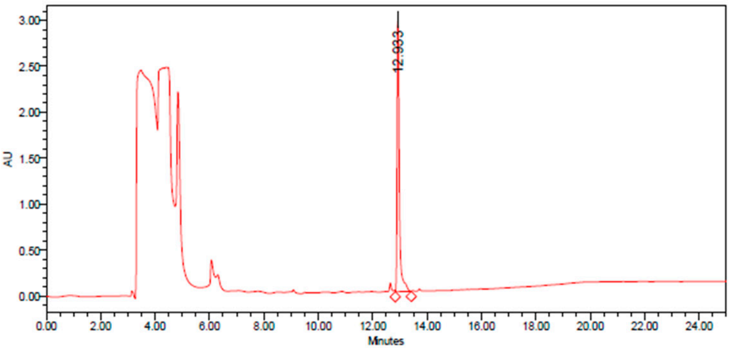

Peak Results

	RT	Area	% Area	Height
1	12.933	17573821	100.00	3040557

Reported by User: walkup
Report Method: WalkUpReport
Report Method ID 1018
Page: 1 of 1

Project Name: MedChem\MedChem2021-04
Date Printed:
4/28/2021
3:08:12 PM America/New_York

Figure S19. Compound 19.

Empower2 software

WalkUpReport

SAMPLE INFORMATION

Sample Name:	R0609-241	Acquired By:	walkup
Sample Type:	Unknown	Sample Set Name:	rajwanaR0609241210423
Vial:	60	Acq. Method Set:	MethodSetAB5T95B15Min
Injection #:	1	Processing Method:	220W280W15MIN
Injection Volume:	10.00 ul	Channel Name:	W2996 220.0nm-2.0
Run Time:	30.0 Minutes	Proc. Chnl. Descr.:	W2996 PDA 220.0 nm at 2.0
Date Acquired:	4/23/2021 10:14:06 AM EST		
Date Processed:	4/23/2021 10:44:19 AM EST		

Auto-Scaled Chromatogram

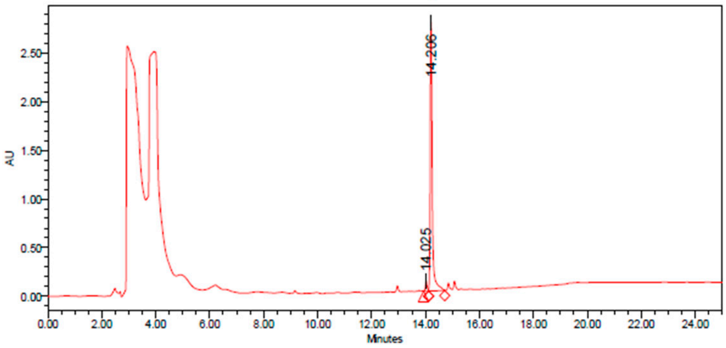

Peak Results

	RT	Area	% Area	Height
1	14.025	463010	3.03	104944
2	14.206	14834915	96.97	2835618

Reported by User: walkup
Report Method: WalkUpReport
Report Method ID: 1018
Page: 1 of 1

Project Name: MedChem\MedChem2021-04
Date Printed:
4/23/2021
10:55:50 AM America/New_York

Figure S20. Compound 20.

Empower 2 software

WalkUpReport

SAMPLE INFORMATION

Sample Name:	R06039-243	Acquired By:	walkup
Sample Type:	Unknown	Sample Set Name:	RajwanaR06039210423
Vial:	98	Acq. Method Set:	MethodSetAB5T95B15Min
Injection #:	1	Processing Method:	220W280W15MIN
Injection Volume:	10.00 ul	Channel Name:	W2996 280.0nm-4.0
Run Time:	30.0 Minutes	Proc. Chnl. Descr.:	W2996 PDA 280.0 nm at 4.0
Date Acquired:	4/23/2021 3:29:39 PM EST		
Date Processed:	4/23/2021 3:59:51 PM EST		

Auto-Scaled Chromatogram

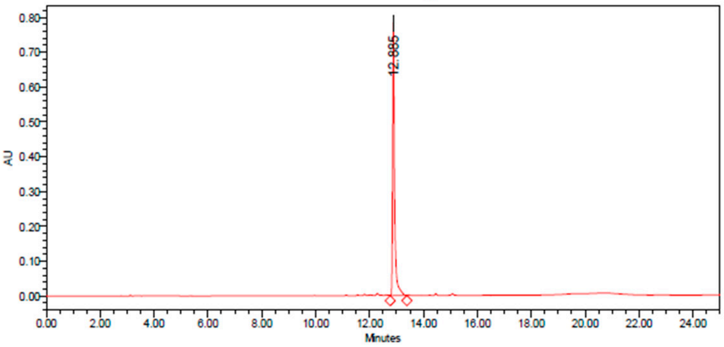

Peak Results

	RT	Area	% Area	Height
1	12.885	3762482	100.00	756826

Reported by User: walkup
Report Method: WalkUpReport
Report Method ID 1018
Page: 1 of 1

Project Name: MedChem\MedChem2021-04
Date Printed:
4/28/2021
2:15:36 PM America/New_York

Figure S21. Compound 21.

Empower2 software

WalkUpReport

SAMPLE INFORMATION

Sample Name:	R06039-244	Acquired By:	walkup
Sample Type:	Unknown	Sample Set Name:	RajwanaR06039
Vial:	46	Acq. Method Set:	MethodSetAB5T95B15Min
Injection #:	1	Processing Method:	220W280W15MIN
Injection Volume:	10.00 ul	Channel Name:	W2996 280.0nm-4.0
Run Time:	30.0 Minutes	Proc. Chnl. Descr.:	W2996 PDA 280.0 nm at 4.0
Date Acquired:	4/29/2021 3:52:00 PM EST		
Date Processed:	4/29/2021 4:22:12 PM EST		

Auto-Scaled Chromatogram

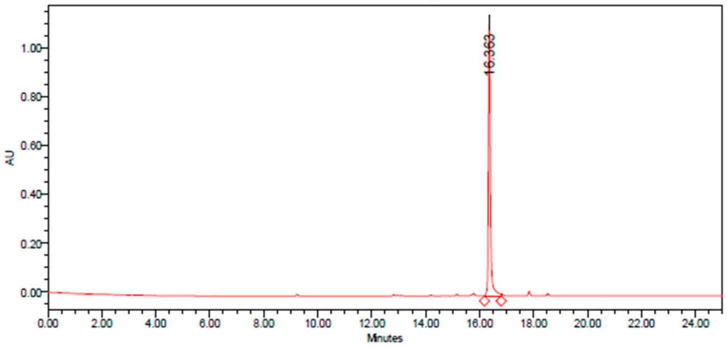

Peak Results

	RT	Area	% Area	Height
1	16.363	5678716	100.00	1113265

Reported by User: walkup
Report Method: WalkUpReport
Report Method ID 1018
Page: 1 of 1

Project Name: MedChem\MedChem2021-04
Date Printed:
4/30/2021
4:00:54 PM America/New_York

Figure S22. Compound 22.

Empower2 software

WalkUpReport

S A M P L E   I N F O R M A T I O N

Sample Name:	R06039-262	Acquired By:	walkup
Sample Type:	Unknown	Sample Set Name:	RajwanaR06039210423
Vial:	102	Acq. Method Set:	MethodSetAB5T95B15Min
Injection #:	1	Processing Method:	220W280W15MIN
Injection Volume:	10.00 ul	Channel Name:	W2996 280.0nm-4.0
Run Time:	30.0 Minutes	Proc. Chnl. Descr.:	W2996 PDA 280.0 nm at 4.0
Date Acquired:	4/23/2021 5:33:29 PM EST		
Date Processed:	4/23/2021 6:03:42 PM EST		

Auto-Scaled Chromatogram

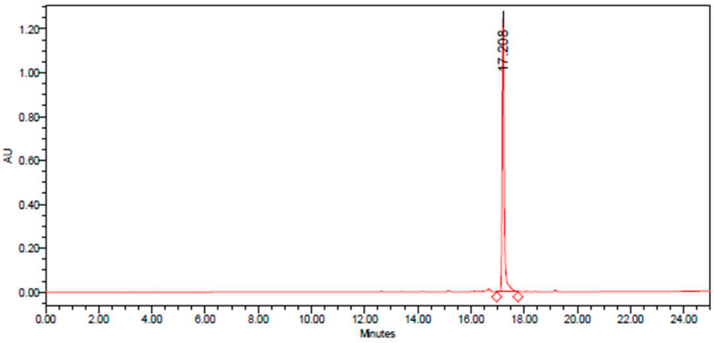

Peak Results

	RT	Area	% Area	Height
1	17.208	6614813	100.00	1245134

Reported by User: walkup
Report Method: WalkUpReport
Report Method ID: 1018
Page: 1 of 1

Project Name: MedChem\MedChem2021-04
Date Printed:
4/28/2021
2:25:14 PM America/New_York

Figure S23. Compound 23.

Empower2 software

WalkUpReport

S A M P L E   I N F O R M A T I O N

Sample Name:	R06039-268	Acquired By:	walkup
Sample Type:	Unknown	Sample Set Name:	RajwanaR06039210423
Vial:	104	Acq. Method Set:	MethodSetAB5T95B15Min
Injection #:	1	Processing Method:	220W280W15MIN
Injection Volume:	10.00 ul	Channel Name:	W2996 280.0nm-4.0
Run Time:	30.0 Minutes	Proc. Chnl. Descr.:	W2996 PDA 280.0 nm at 4.0
Date Acquired:	4/23/2021 6:35:23 PM EST		
Date Processed:	4/28/2021 2:28:11 PM EST		

Auto-Scaled Chromatogram

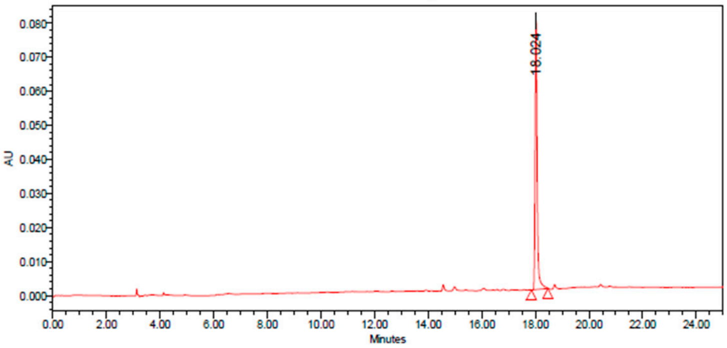

Peak Results

	RT	Area	% Area	Height
1	18.024	417386	100.00	79381

Reported by User: walkup
Report Method: WalkUpReport
Report Method ID: 1018
Page: 1 of 1

Project Name: MedChem\MedChem2021-04
Date Printed:
4/28/2021
2:53:25 PM America/New_York

Figure S24. Compound 24.

Empower2 software

WalkUpReport

SAMPLE INFORMATION

Sample Name:	R06039-242	Acquired By:	walkup
Sample Type:	Unknown	Sample Set Name:	RajwanaR06039210423
Vial:	97	Acq. Method Set:	MethodSetAB5T95B15Min
Injection #:	1	Processing Method:	220W280W15MIN
Injection Volume:	10.00 ul	Channel Name:	W2996 220.0nm-2.0
Run Time:	30.0 Minutes	Proc. Chnl. Descr.:	W2996 PDA 220.0 nm at 2.0
Date Acquired:	4/23/2021 2:58:40 PM EST		
Date Processed:	4/28/2021 2:55:20 PM EST		

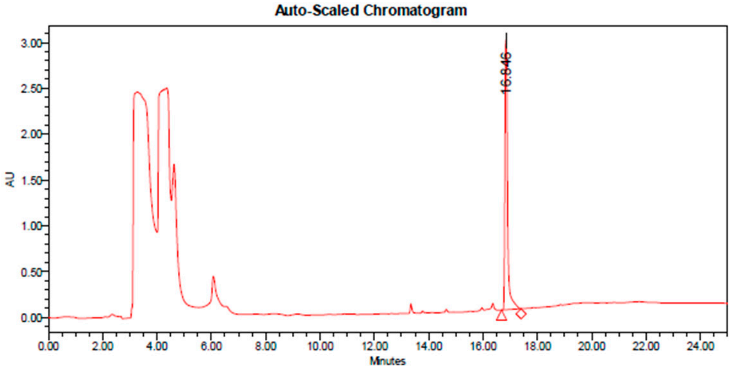

Peak Results

	RT	Area	% Area	Height
1	16.846	20121130	100.00	2986796

Reported by User: walkup
Report Method: WalkUpReport
Report Method ID: 1018
Page: 1 of 1

Project Name: MedChem\MedChem2021-04
Date Printed:
4/28/2021
3:05:24 PM America/New_York

Figure S25. Compound 25.

WalkUpReport

SAMPLE INFORMATION

Sample Name:	R06039-238	Acquired By:	walkup
Sample Type:	Unknown	Sample Set Name:	RajwanaR06039210423
Vial:	13	Acq. Method Set:	MethodSetAB5T95B15Min
Injection #:	1	Processing Method:	220W280W15MIN
Injection Volume:	10.00 ul	Channel Name:	W2996 220.0nm-2.0
Run Time:	30.0 Minutes	Proc. Chnl. Descr.:	W2996 PDA 220.0 nm at 2.0
Date Acquired:	4/24/2021 4:23:59 AM EST		
Date Processed:	4/28/2021 2:49:58 PM EST		

Auto-Scaled Chromatogram

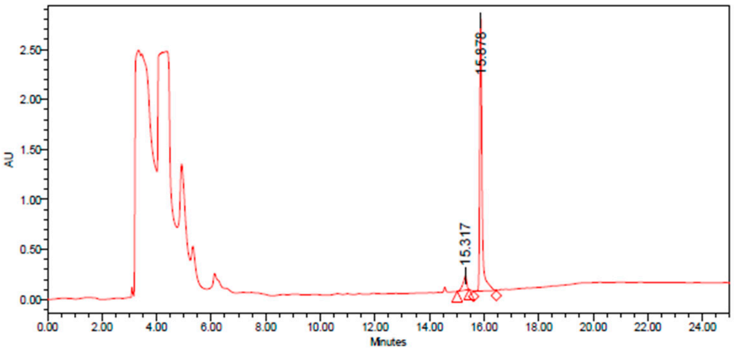

Peak Results

	RT	Area	% Area	Height
1	15.317	1320549	7.46	144081
2	15.878	16391937	92.54	2747838

Reported by User: walkup
Report Method: WalkUpReport
Report Method ID 1018
Page: 1 of 1

Project Name: MedChem\MedChem2021-04
Date Printed:
4/28/2021
3:02:27 PM America/New_York

Figure S26. Compound 26.

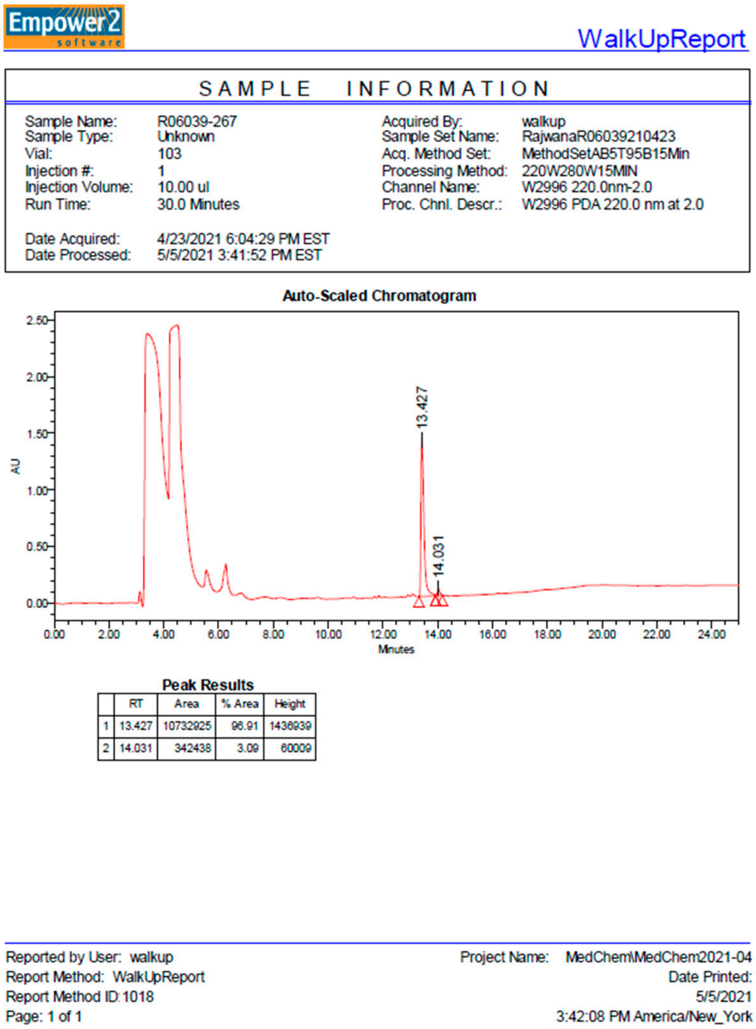

Empower2 software WalkUpReport

SAMPLE INFORMATION

Sample Name:	R06039-267	Acquired By:	walkup
Sample Type:	Unknown	Sample Set Name:	RajwanaR06039210423
Vial:	103	Acq. Method Set:	MethodSetAB5T95B15Min
Injection #:	1	Processing Method:	220W280W15MIN
Injection Volume:	10.00 ul	Channel Name:	W2996 220.0nm-2.0
Run Time:	30.0 Minutes	Proc. Chnl. Descr.:	W2996 PDA 220.0 nm at 2.0
Date Acquired:	4/23/2021 6:04:29 PM EST		
Date Processed:	5/5/2021 3:41:52 PM EST		

Auto-Scaled Chromatogram

Peak Results

	RT	Area	% Area	Height
1	13.427	10732925	96.91	1436939
2	14.031	342438	3.09	60009

Reported by User: walkup
Report Method: WalkUpReport
Report Method ID: 1018
Page: 1 of 1

Project Name: MedChem\MedChem2021-04
Date Printed:
5/5/2021
3:42:08 PM America/New_York

Figure S27. Compound 27.

Empower2 software

WalkUpReport

SAMPLE INFORMATION

Sample Name:	R06039-237	Acquired By:	walkup
Sample Type:	Unknown	Sample Set Name:	RajwanaR06039210423
Vial:	12	Acq. Method Set:	MethodSetAB5T95B15Min
Injection #:	1	Processing Method:	220W280W15MIN
Injection Volume:	10.00 ul	Channel Name:	W2996 220.0nm-2.0
Run Time:	30.0 Minutes	Proc. Chnl. Descr.:	W2996 PDA 220.0 nm at 2.0
Date Acquired:	4/24/2021 3:53:01 AM EST		
Date Processed:	4/28/2021 2:49:21 PM EST		

Auto-Scaled Chromatogram

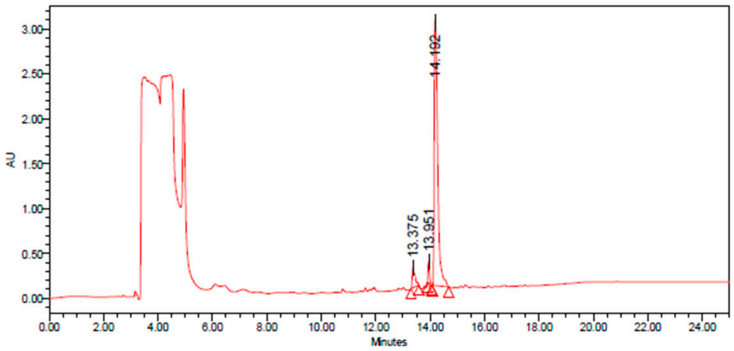

Peak Results

	RT	Area	% Area	Height
1	13.375	1580261	5.52	223486
2	13.951	942261	3.29	237265
3	14.192	26115200	91.19	3051355

Reported by User: walkup
Report Method: WalkUpReport
Report Method ID: 1018
Page: 1 of 1

Project Name: MedChem\MedChem2021-04
Date Printed:
4/28/2021
3:01:52 PM America/New_York

Figure S28. Compound 28.

WalkUpReport

SAMPLE INFORMATION

Sample Name:	R06039-257	Acquired By:	walkup
Sample Type:	Unknown	Sample Set Name:	RajwanaR06039
Vial:	48	Acq. Method Set:	
Injection #:	1	Processing Method:	220W280W15MIN
Injection Volume:	20.00 ul	Channel Name:	W2996 220.0nm-2.0
Run Time:	30.0 Minutes	Proc. Chnl. Descr.:	W2996 PDA 220.0 nm at 2.0
Date Acquired:	4/29/2021 4:54:02 PM EST		
Date Processed:	4/30/2021 4:03:53 PM EST		

Auto-Scaled Chromatogram

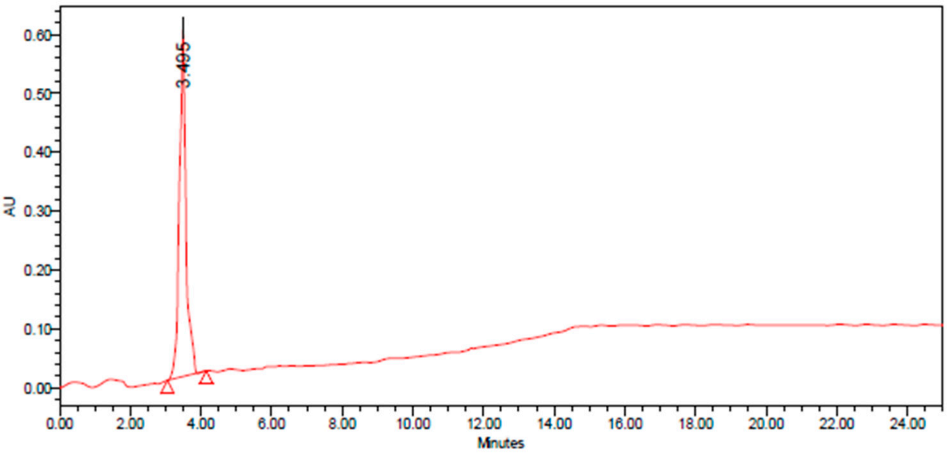

Peak Results

	RT	Area	% Area	Height
1	3.495	7635741	100.00	591338

Reported by User: walkup
Report Method: WalkUpReport
Report Method ID: 1018
Page: 1 of 1

Project Name: MedChem\MedChem2021-04
Date Printed:
4/30/2021
4:08:08 PM America/New_York

Figure S29. Compound 29.
